# *Datura* quids at Pinwheel Cave, California, provide unambiguous confirmation of the ingestion of hallucinogens at a rock art site

**DOI:** 10.1073/pnas.2014529117

**Published:** 2020-11-23

**Authors:** David W. Robinson, Kelly Brown, Moira McMenemy, Lynn Dennany, Matthew J. Baker, Pamela Allan, Caroline Cartwright, Julienne Bernard, Fraser Sturt, Elena Kotoula, Christopher Jazwa, Kristina M. Gill, Patrick Randolph-Quinney, Thomas Ash, Clare Bedford, Devlin Gandy, Matthew Armstrong, James Miles, David Haviland

**Affiliations:** ^a^School of Forensic and Applied Sciences, University of Central Lancashire, PR1 2HE Preston, United Kingdom;; ^b^WestCHEM, Department of Pure and Applied Chemistry, University of Strathclyde, G1 1RD Glasgow, United Kingdom;; ^c^Department of Scientific Research, British Museum, WC1B 3DG London, United Kingdom;; ^d^Department of Anthropology, Geography, and Geology, East Los Angeles Community College, Monterey Park, CA 91754;; ^e^Department of Archaeology, University of Southampton, SO17 1BJ Southampton, United Kingdom;; ^f^Information Services Group, University of Edinburgh, EH8 9YL Edinburgh, United Kingdom;; ^g^Human Paleoecology and Archaeometry Laboratory, Department of Anthropology, University of Nevada, Reno, NV 89557-0096;; ^h^Museum of Natural and Cultural History, University of Oregon, Eugene, OR 97403;; ^i^Department of Applied Sciences, Faculty of Health and Life Sciences, Northumbria University, NE1 8ST Newcastle Upon-Tyne, United Kingdom;; ^j^Department of Human Anatomy and Physiology, Faculty of Health Sciences, University of Johannesburg, Aukland Park 2006, South Africa;; ^k^Department of Archaeology, University of Cambridge, CB2 3DZ Cambridge, United Kingdom;; ^l^Pacific Gas and Electric Company, Fresno, CA 93710;; ^m^Archaeovision UK, Chesham, HP5 3DQ, United Kingdom;; ^n^University of California Cooperative Extension, Bakersfield, CA 93307

**Keywords:** hallucinogens, rock art, *Datura*, quids, Native California

## Abstract

While debates have raged over the relationship between trance and rock art, unambiguous evidence of the consumption of hallucinogens has not been reported from any rock art site in the world. A painting possibly representing the flowers of *Datura* on the ceiling of a Californian rock art site called Pinwheel Cave was discovered alongside fibrous quids in the same ceiling. Even though Native Californians are historically documented to have used *Datura* to enter trance states, little evidence exists to associate it with rock art. A multianalytical approach to the rock art, the quids, and the archaeological context of this site was undertaken. Liquid chromatography−mass spectrometry (LC-MS) results found hallucinogenic alkaloids scopolamine and atropine in the quids, while scanning electron microscope analysis confirms most to be *Datura wrightii*. Three-dimensional (3D) analyses of the quids indicate the quids were likely masticated and thus consumed in the cave under the paintings. Archaeological evidence and chronological dating shows the site was well utilized as a temporary residence for a range of activities from Late Prehistory through Colonial Periods. This indicates that *Datura* was ingested in the cave and that the rock painting represents the plant itself, serving to codify communal rituals involving this powerful entheogen. These results confirm the use of hallucinogens at a rock art site while calling into question previous assumptions concerning trance and rock art imagery.

## Significance

Proponents of the altered states of consciousness (ASC) model have argued that hallucinogens have influenced the prehistoric making of images in caves and rock shelters. However, the lack of direct evidence for the consumption of hallucinogens at any global rock art site has undermined the ASC model. We present the first clear evidence for the ingestion of hallucinogens at a rock art site, in this case, from Pinwheel Cave, California. Quids in the cave ceiling are shown to be *Datura wrightii*, a Native Californian entheogen, indicating that, rather than illustrating visual phenomena caused by the *Datura*, the rock paintings instead likely represent the plant and its pollinator, calling into question long-held assumptions about rock art and the ASC model.

Since the late 1980s, the role that altered states of consciousness (or ASC) played in the making of rock art has been one of the most contentious questions confronted by rock art researchers across the globe (1–7). The ASC model purports that humans universally experience three distinct visual phases during trance, which are replicated in rock art imagery (1). The ASC model can be induced in a number of ways including the use of hallucinogenic substances (1). However, there remains no clear evidence for the preparation and consumption of hallucinogenic substances directly associated with any rock art site in the world. Indeed, fierce debate has occurred over the last 30 y, with many researchers questioning the validity of the ACS model and the idea of shamanism as a viable explanation for the creation of rock art (2–6). California has been central within this debate (8, 9). Whitley (9) has argued that the many south-central Californian rock paintings were shamanic self-portraits depicting a shaman’s experience during ASC while rock art sites were owned by individual shamans, and avoided by the local populace. In this view, trance, shamanism, and rock art are inextricably linked in their separation from normal activity of the wider populace. However, evidence from systematic archaeological work in south-central California has clearly shown that the majority of rock art sites were integrated into habitation sites, and are not separated from public view (10, 11). Recent analyses also suggest that the pictographs were probably not self-depictions of shamans in trance but, instead, stock iconographic images drawing upon mythology and the personifying of insects, animals, plant, and astronomical elements such as the sun (12, 13).

Even so, ethnographic documentation details how hallucinogens played a pivotal role in Native California, especially *Datura wrightii* (14). A member of the Solanacae family, *Datura* is distinctive by large white “trumpet” flowers that uncoil in a five-pointed pinwheeling fashion. *Datura* as a genus can be found across multiple continents, including the Americas, Asia, Europe, and South Africa (15). Its wide availability and hallucinogenic properties, due to the presence of the tropane alkaloids atropine and scopolamine, are behind its use across different cultures (16, 17). The most noted usage of *Datura* in Native California is in youth initiations where the root was processed into a drink or “tea” known historically as toloache (14, 18–21). Initiates would often be instructed in cultural rules of entering adulthood and how to interpret the visions themselves (14, 19, 21). For some, these ceremonies where highly codified, such as the Chinigchinich religion of Southern California, which ended with the making of a sand painting in which boys learned religious principles (19, 21). The sand paintings did not depict the visions induced by *Datura*, but, instead, were cosmological maps detailing the ontological principles of the Southern Californian Native societies making them (22). After undergoing the puberty ceremony, *Datura* could be taken throughout one’s lifetime for a variety of reasons, including to gain supernatural power for doctoring, to counteract negative supernatural events, to ward off ghosts, and to see the future or find lost objects, but, most especially, as a mendicant for a variety of ailments (14, 18, 23). *Datura* consumption could occur prior to hunting to increase stamina and power (24). Importantly, *Datura* could be consumed in a variety of ways, including drinking toloache, but also by roasting the roots, eating the flowers or seeds, applying poultices on wounds, or often simply chewing the roots or other parts of the plant (14). For the Tübatulabal, *Datura* originated as a man named *Mo mo ht* who subsequently turned into the plant in its present form (25), while, in Chumash mythology, the plant was a prominent supernatural grandmother called *Momoy (*26). Since *Datura*, with its psychoactive substances, was used within spiritual, ritual, and mythological contexts, it should be considered as an entheogen (27).

Worldwide, different hallucinogens have been suggested as inspiring rock art making, such as mushrooms, Peyote, *Datura*, San Pedro cactus, *Brunsvigia*, and others (2, 28–35). *Datura* is of particular focus in the North American West. Malotki (33) argues that *Datura* influenced archaic Basketmaker rock art in Arizona, while Boyd’s (32) extensive analyses have shown that Lower Pecos rock art iconography likely relates to mythological narratives concerning Peyote and *Datura*. Images include hornworms, the larval stages of hawkmoths, *Datura’s* primary pollinator. Mimbres pottery and Kiva murals also include representations of *Datura*, plus anthropomorphized versions of the hawkmoth (36, 37). Evidence of *Datura* alkaloids have been found in ceramics (38), while ancient peyote buttons and *Datura* seeds have been found in Lower Pecos archaeological deposits, but none have been reported specifically at rock art sites (39).

**Fig. 1.**
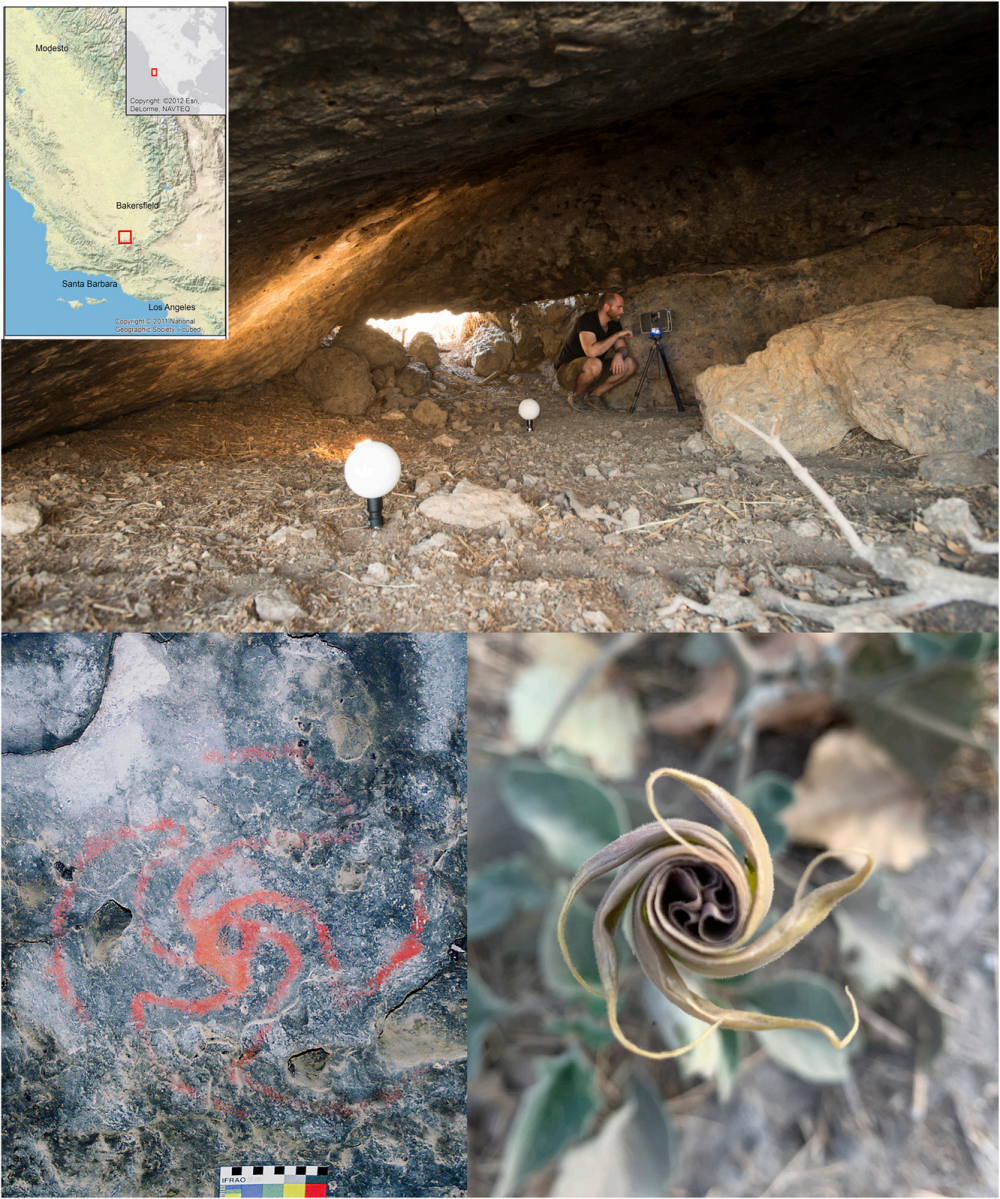
Pinwheel Cave, California. (*Top*) Interior of cave during laser scanning. (*Bottom Left*) Pinwheel painting within cave. Image credit: Rick Bury (photographer). (*Bottom Right*) Unfurling flower of *D. wrightii* from plant near cave site. Image credit: Melissa Dabulamanzi (photographer).

*Datura* has also been suggested as relating to the making of Chumash rock art in California (40). Found in the Chumash borderlands of interior south-central California, we detail here our investigations at a rock art site called Pinwheel Cave (CA-KER-5836) (Fig. 1) and its associated food processing bedrock mortar (BRM) complex (CA-KER-5837). The name originates from a large red pinwheel motif, which we hypothesized may represent the opening *Datura* flower (Fig. 1, *Bottom Left* and *Bottom Right*). Dozens of fibrous clumps known as quids are also located within crevices in the cave ceiling (41). Quids, usually found in archaeological deposits, are typically made of yucca, agave, tule, or tobacco and are thought to have been chewed to extract nutrients or stimulants (41–44). With the painting likely representing the opening of the *Datura* flower, and with the very unusual insertion of quids in the ceiling, we investigated the possibility that the quids could contain *Datura*. We present the results of a multianalytical investigation of the quids and the archaeological context to investigate the potential use of *Datura* in association with rock art iconography.

## Results

### Quid Three-Dimensional Digital Microscopy.

**Fig. 2.**
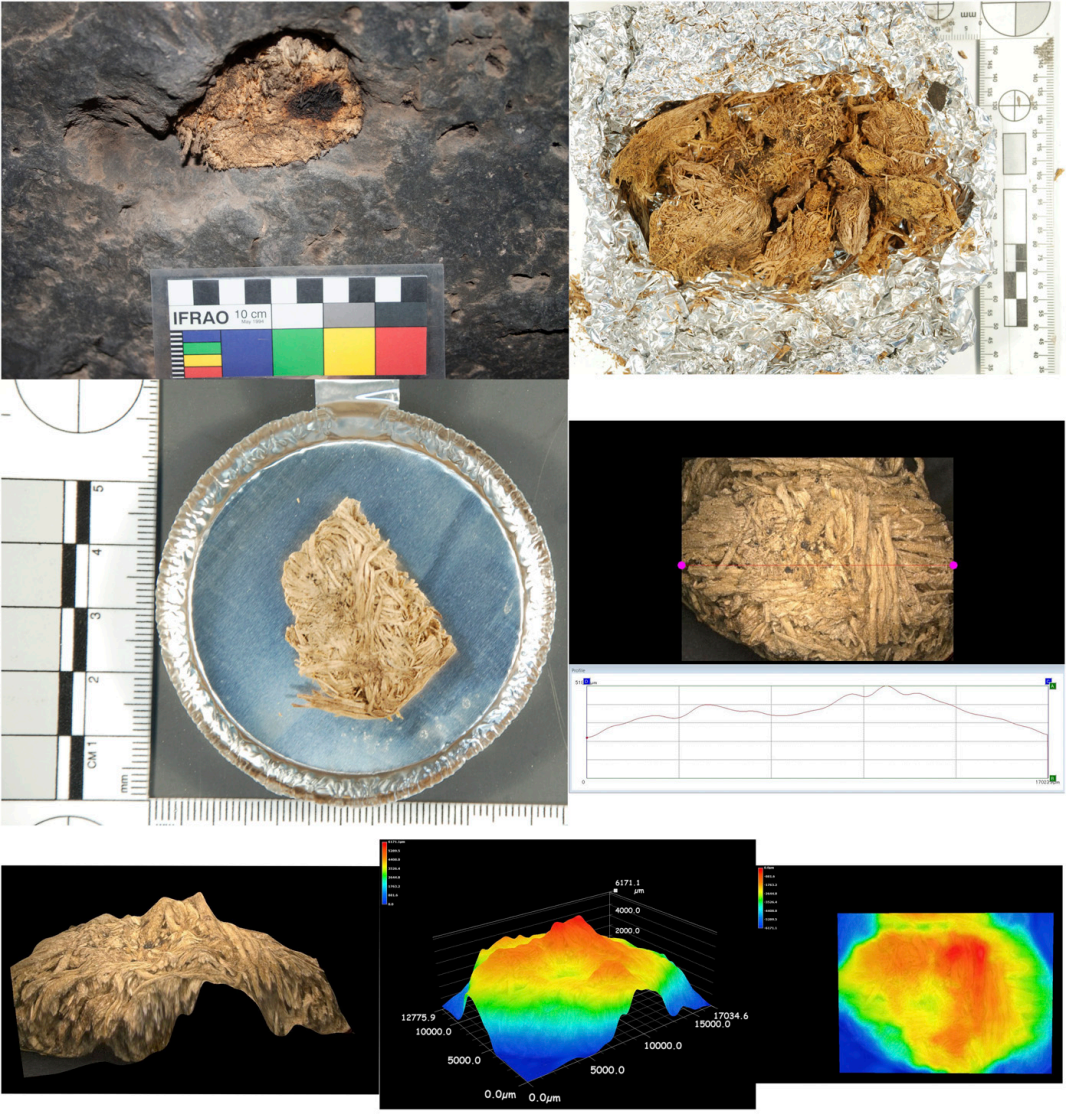
Quid analyses. (*Top Left*) Quid sample 2 in situ in ceiling before removal. (*Top Right*) Quid sample 2 after removal of surrounding husk showing multiple quids. (*Middle Left*) Quid 2, subsample 5 in isolation, dorsal view. (*Middle Right*) Horizontal topographic reading of subsample 5, dorsal side. (*Bottom Left*) A 3D view of subsample 5, dorsal side. (*Bottom Middle*) A 3D heat map of subsample 5, dorsal side. (*Bottom Right*) A 2D heat map subsample 5, dorsal side.

**Fig. 3.**
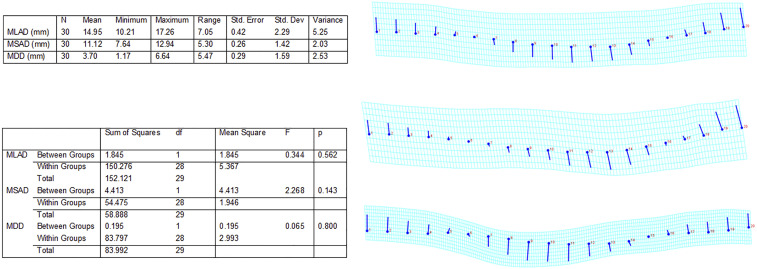
GMM shape analysis. (*Top Left*) Table of descriptive statistics for dimensions of quid surface depressions. (*Bottom Left*) Table of one-way ANOVA of depression metric measurements comparing dorsal and ventral surface dimensions. (*Right*) Transformation grids of coordinates of quid surfaces following GPA, with subsequent PCA based on Procrustes coordinates. All transformations and visualizations were undertaken in MorphoJ. Each grid shows variation on the first principal axis of variation (PC1). (*Top*) The overall pattern of variation in the sample (*n* = 30, LM = 20; PC1 accounts for 48.5% of overall variation), (*Middle*) patterns of variation in the dorsal surface depressions (*n* = 15, LM = 20; PC1 accounts for 61.2% of variation in dorsal surface shape), and (*Bottom*) the pattern of variation in ventral surface depressions (*n* = 15, LM = 20; PC1 accounts for 44.2% of variation in ventral surface shape). All three profiles are broadly consistent, presenting an internal depressed concavity with raised lateral margins.

Imaging clearly shows that the fibrous materials constituting the quids are crushed and matted together in tightly compact clumps (Fig. 2) (45) which display a topography marked by depressions on one or more of the exposed surfaces. In order to first assess whether the quids had been masticated (43) and may therefore have been orally processed, a three-dimensional (3D) digital microscopy analysis was undertaken on 15 quids to examine their topography and potential tooth depressions (Fig. 2 and *SI Appendix*, Fig. S4) (45). The table in Fig. 3, *Top Left* highlights the basic descriptive statistics of the surface depressions. The measurements were normally distributed, and no statistical difference was noted between the dimensions of the depressions occurring on the dorsal and ventral surfaces following one-way ANOVA (Fig. 3, *Bottom Left*). The 3D visualization and heat map production indicate that both surfaces of each quid present a single variable depression, with raised outer margins and a somewhat variable base (undulating or crenulated). This varies in location—ranging from occurring on or toward the margin of the quid to being more centrally positioned within the body of the substrate (see *SI Appendix* for surface microscopy of each specimen).

Geometric morphometric methods (GMM) statistical shape analysis was informative. The use of Procrustes superimposition (generalized Procrustes analysis [GPA]) ensures that sized-based effects are removed, and only shape-based differences remain. Principal components analysis (PCA) of the total sample yielded 29 principal components with nonzero variability. The first three principal components together accounted for 82.9% of variation, with the first seven factors accounting for around 95% of shape variation in the sample. The resulting average shape configurations of the first principal components for the overall (pooled) sample, and dorsal and ventral subsets, were determined (Fig. 3, *Right*). Each grid shows variation on the first principal axis of variation (PC1). The top grids shows the overall pattern of variation in the sample (*n* = 30, Landmarks [LM] = 20; PC1 accounts for 48.5% of overall variation), the middle grid shows patterns of variation in the dorsal surface (*n* = 15, LM = 20; PC1 accounts for 61.2% of variation in dorsal surface shape), and the bottom grid shows the pattern of variation in the ventral surface (*n* = 15, LM = 20; PC1 accounts for 44.2% of variation in ventral surface shape). All three profiles are broadly consistent, presenting an internal depressed concavity with raised lateral margins. Shape and size homogeneity for surface depressions within the sample is supported by Procrustes ANOVA tests on both size (as reflected by centroid) and shape. Procrustes ANOVA of both size and shape residuals indicated no significant size or shape differences within the dorsal and ventral surface of the samples (centroid only F = 2.88, *P* = 0.1009; shape only F = 1.07, *P* = 0.3595), supporting homogeneity within the surface depressions. This homogeneity suggests a similar mechanism for the production of the ventral or dorsal depressions. The highly fibrous nature of the quids precludes the detailed preservation of tooth occlusal surface (cusp) morphology as seen in other studies (43). However, it is clear that each quid analyzed had been bilaterally compressed, leading to the formation of a depression on both opposing surfaces; the most parsimonious interpretation is that the quids were compressed between the occlusal surfaces of the upper and lower dentition, with masticatory loading (bite force) causing the formation of ventral and dorsal depressions.

### Liquid Chromatography−Mass Spectrometry Study.

**Fig. 4.**
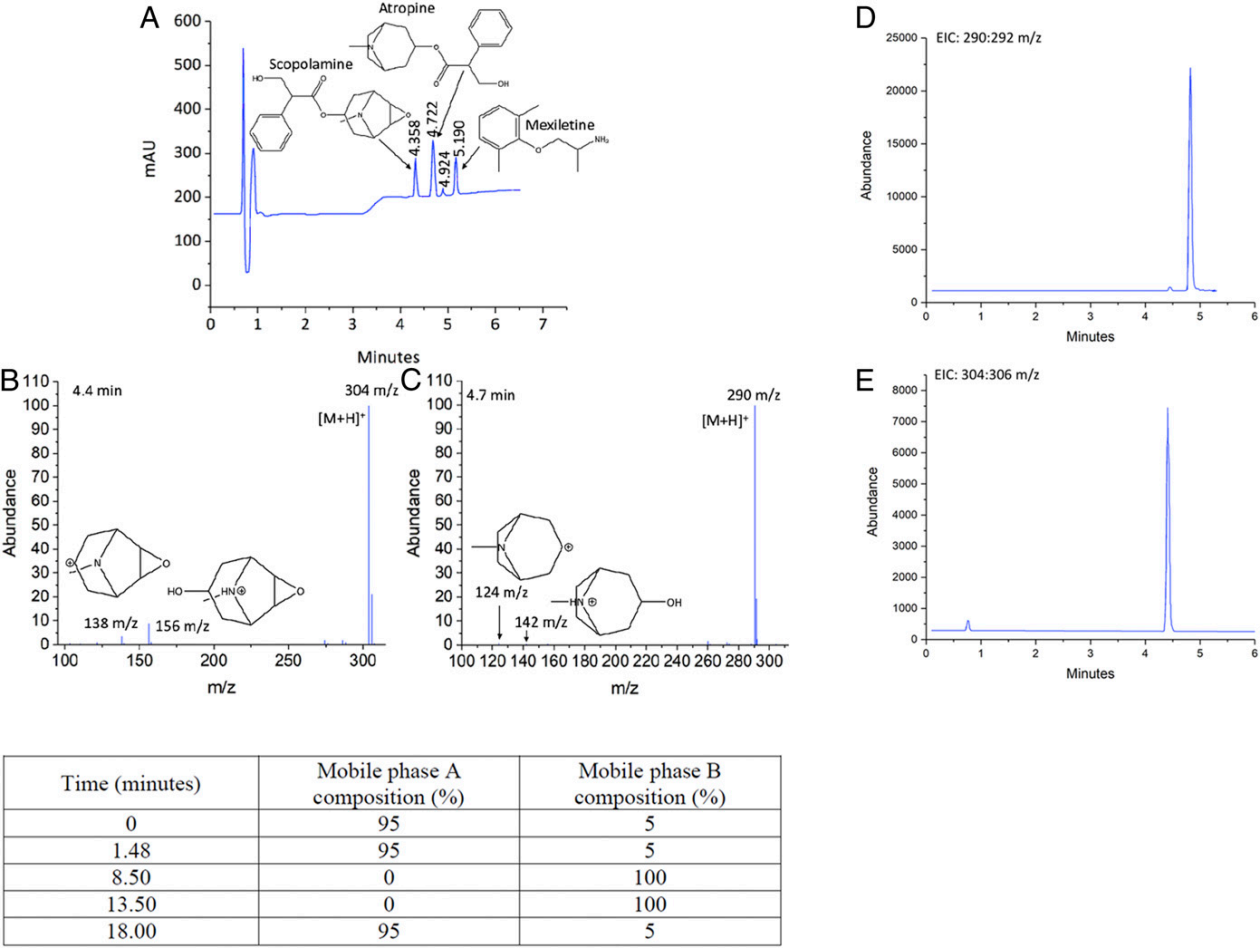
LC-MS analyses of quids. (*A−C*) Chromatographic and mass spectrometry data of mixed tropane alkaloid standard. (*A*) Chromatographic analysis of a mixed standard at concentrations of 0.2 mg⋅mL^−1^ for atropine and scopolamine and 0.05 mg⋅mL^−1^ for mexiletine recorded at 214 nm. (*B*) Mass spectra of scopolamine taken across a time range of 4.384 min to 4.477 min, with important fragment peaks highlighted. (*C*) Mass spectra of atropine taken across a time range of 4.755 min to 4.848 min, with important fragment peaks highlighted. (*D* and *E*) Extracted ion chromatograms for Kerr 5832 subsample 4, replicate 2. (*D*) EIC of atropine over 290 to 292 *m/z* range. (*E*) EIC of scopolamine over 304 to 306 *m/z* range. (*Bottom*) Table of LC-MS gradient flow profile details, where mobile phase A was 0.1%vol/vol formic acid water and mobile phase B was 0.1%vol/vol formic acid acetonitrile.

As a first phase, identification of the tropane alkaloids atropine and scopolamine was attempted to tentatively determine whether *Datura* was indeed present within 4 of the 15 quid samples recovered from the cave. Extraction of both the quids and dried *Datura* leaf material, utilized as a control, was performed to extract any atropine and scopolamine present within the solid material. Both acidic (46–49) and alkaline-based extraction (50, 51) methods utilizing a range of solvents have previously been performed to varying degrees of success for the extraction of the tropane alkaloids from leaf (48, 49), flower (47), root (51), seeds (47, 49, 50, 52), and stem (47, 49) material of *Datura* plants. A set of mixed standards containing atropine, scopolamine, and mexiletine hydrochloride, utilized as an internal standard, prepared within the extraction solvent methanol, were analyzed by reverse phase liquid chromatograph−mass spectrometry (LC-MS) with both ultraviolet (UV) (214-nm detector wavelength) and mass spectrometry detection. Analysis of the standards provided confidence in the chromatographic method for successful identification of all three analytes when present within a single sample matrix. With atropine and scopolamine the dominant hallucinogenic tropane alkaloids present within *Datura,* they were chosen as the analytes of interest for identification within the quid samples. Analytes were positively identified through comparison of retention times to pure standards alongside their pseudomolecular ion fragments at 290 and 304 *m/z* for atropine and scopolamine, respectively. Further confirmation of the presence of the analytes could be made through their additional ion fragmentation patterns. These include fragments at both 138 and 156 *m/z* for scopolamine, where the fragment at 138 *m/z* with the molecular formula [C_8_H_12_NO]^+^ is a result of the loss of tropic acid and the fragment at 156 *m/z* is the result of the loss of the neutral fragment C_9_H_8_O_2_, due to the cleavage of the C−O bond between the carbonyl carbon of the ester group and the oxygen of the ether group and has the molecular formula [C_8_H_14_NO_2_]^+^ (Fig. 4*B*). Atropine fragments were observed at 124 and 142 *m/z*, where the fragment at 124 *m/z* with a molecular formula of [C_8_H_14_N] ^+^ similar to scopolamine is due to the loss of tropic acid and the fragment at 142 *m/z* is the result of C−O bond cleavage between the carbonyl carbon and oxygen of the ester group and has the molecular formula [C_8_H_16_NO]^+^ (Fig. 4 *C*, *Left*). Analysis of the pure standards revealed these fragments were observed in low abundance compared with their pseudomolecular ion, as such identification within quids would be primarily made through identification of the pseudomolecular ion fragment rather than their additional ion fragments. The chromatographic analysis showed well-separated peaks with no coelution (Fig. 4 *A*, *Left*) and resolution values all greater than one. Atropine and mexiletine produced only one significant peak within the UV chromatogram; however, the scopolamine reference standard produced a number of additional impurity peaks. As these peaks were not found within the pure reference standards of atropine or mexiletine or within the methanol blanks, they were intrinsic to the scopolamine reference standard and not a result of instrument contamination. Their presence can likely be accounted for due to the lower purity of the scopolamine reference standard at ≥90% compared with ≥99% for atropine and mexiletine. As the impurity peaks did not interfere with any of the analytes of interest and were not observed within the mass spectrum, they did not negatively impact the analysis and could be discounted with confidence.

### LC-MS Analysis of Quids.

Atropine and scopolamine were firmly identified within the four quid samples analyzed; Ker-5836 subsamples two, four, five, and nine. The extracted ion chromatograms (EIC) for each tropane alkaloid found within Ker-5836 subsample four replicate two are shown in Fig. 4 *D* and *E*. All chromatographic and mass spectra data for subsamples two (replicates one and two), four (replicates one and two), five, and nine are included within *SI Appendix*, Fig. S6. As a result of the low concentration of the tropane alkaloids within the quids, the total iron current (TIC) could not be used for the reliable detection of the analytes. The mass of the pseudomolecular ion was therefore used to construct EIC to identify whether the tropane alkaloids were indeed present within the quids. Results highlight the variations in the concentration of both of the alkaloids present between replicates taken from the same quid sample in addition to variations between quids. A general trend was identified among the majority of the quids, with atropine detected at an apparently higher concentration than scopolamine, in all bar one replicate of subsample four, while, in subsample nine, only atropine was successfully detected. These results were not unexpected, with variations in the concentration of both alkaloids between different species of *Datura*, plants of the same species, plant material, and times of plant harvesting all previously reported (16, 17, 47, 49, 53–55). Furthermore, Śramska et al. (55) demonstrated similar variation within the concentrations of both alkaloids within different portions taken from the same parent plant. They concluded that the complex removal of the alkaloids from within the plant cells contributes to the poor reproducibility observed for plant samples, despite achieving good reproducibility with their extraction procedure within control samples (55). As such, this complexity in extraction has likely contribution to the variations observed here.

With atropine and scopolamine the two dominant tropane alkaloids found within *Datura*, their presence within the analyzed quids alongside the scanning electron microscope (SEM) analysis strongly indicates they are formed, in some part, from the *Datura* plant. However, it must be noted other plant species could also contribute to their presence including, *Atropa Belladonna, Brugmansia*, and *Scopolia.*

### SEM Results.

**Fig. 5.**
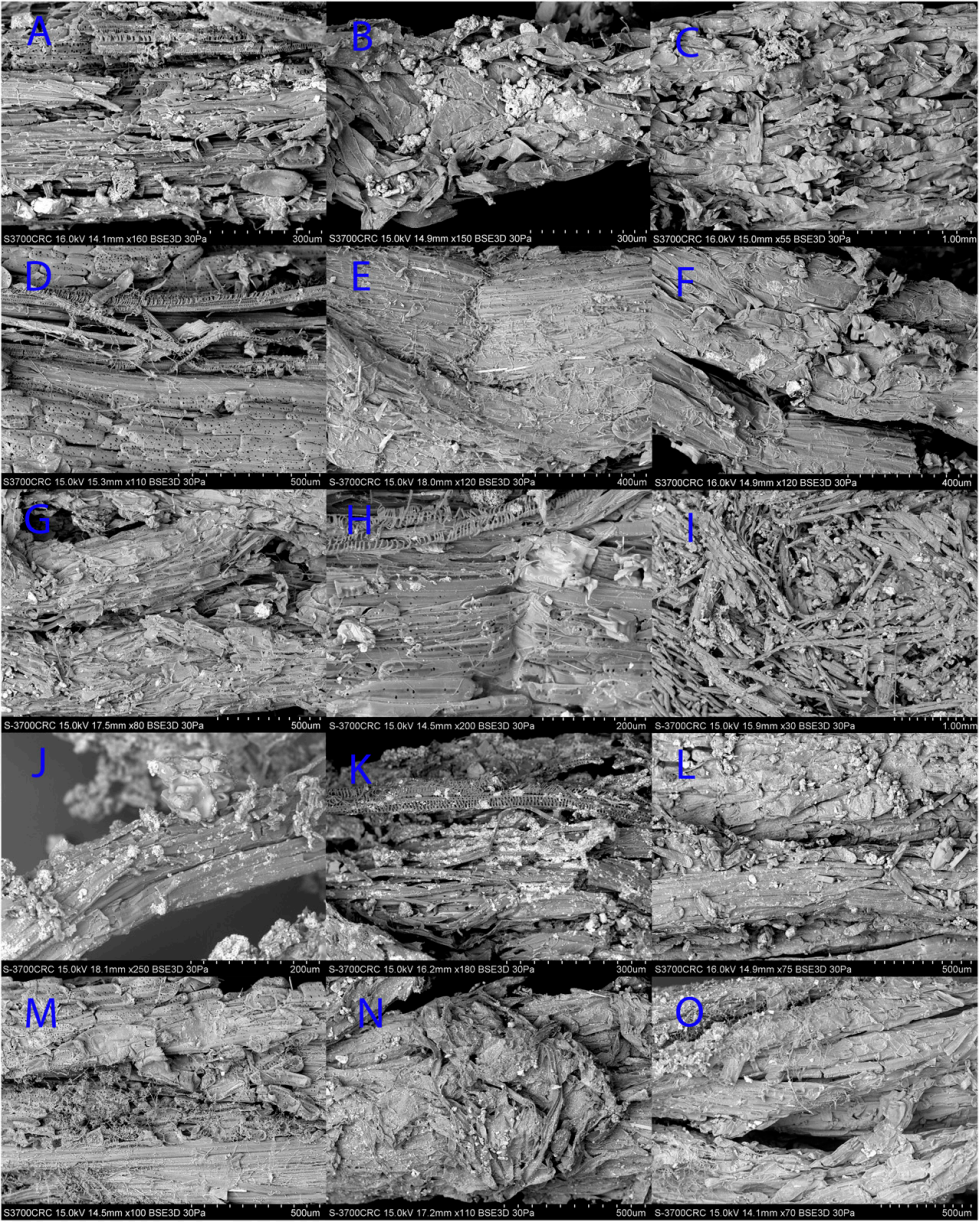
SEM identification of the quids. (*A*) Quid 1, *D. wrightii*; (*B*) quid 2, subsample 1, *D. wrightii*; (*C*) quid 2, subsample 3, *D. wrightii*; (*D*) quid 2, subsample 4, *D. wrightii*; (*E*) quid 2, subsample 5, *D. wrightii*; (*F*) quid 2, subsample 7, *D. wrightii*; (*G*) quid 2, subsample 8, *D. wrightii*; (*H*) quid 2, subsample 9, *D. wrightii*; (*I*) quid 2, subsample 10, *D. wrightii*; (*J*) quid B, *Yucca*; (*K*) quid C, *D. wrightii*; (*L*) quid E, *D. wrightii*; (*M*) quid F, *D. wrightii*; (*N*) quid G, *D. wrightii*; and (*O*) quid H, *D. wrightii*.

Following the successful identification of hallucinogenic alkaloids in the quids outlined above, all 15 quids recovered from the cave were SEM identified. As a result of the SEM examination, genus-level identifications was possible. Fig. 5 shows that almost all of the samples are *D. wrightii*, with the one exception being *Yucca* sp.—quid B (Fig. 5*J*). The four samples from the LC-MS study were verified as *Datura*. As can be seen in the SEM images, the quids show evidence of having been crushed, which is consistent with the GMM shape analysis above. Inclusions within the quids also reflect that they were inserted in the cave ceiling; some are sandy (e.g., quid G; Fig. 5*N*), and many have calcareous particles present. Fungal hyphae and spores were visible at magnifications of 100× and above in the SEM, especially, noticeable in quid F (Fig. 5*M*). Different parts of plants were present, but most were tiny, cominuted leaf fragments, and also some stem fragments. As the SEM examination was conducted on samples and subsamples of quids, it is impossible to quantify relative proportions of particles.

### Archaeological Investigations at Pinwheel Cave and BRM Complex.

**Fig. 6.**
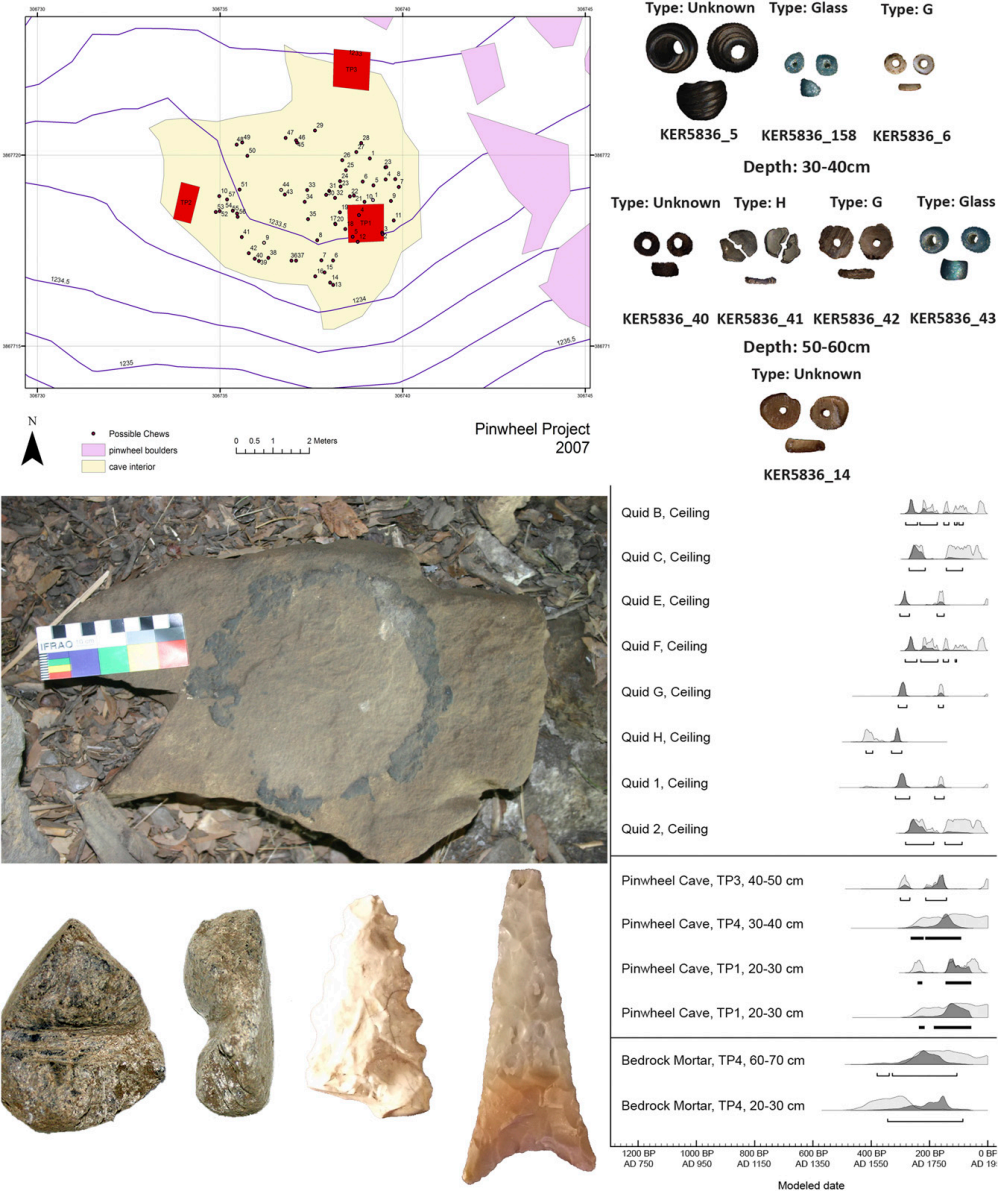
Archaeology and dating of Pinwheel Cave. (*Top Left*) Map of cave with location of identifiable quids. (*Top Right*), Example of beads. (*Middle Left*) Hopper mortar on surface of cave deposits. (*Middle Right*) Accelerator mass spectrometry dates from Pinwheel Cave and the BRM complex; light gray are unmodeled dates, and dark gray are modeled dates. (*Bottom, Left* to *Right*) Top view of arrow shaft straightener, side view of arrow shaft straightener, portion of serrated projectile point, and Cottonwood triangular projectile point.

There are three openings into the Main Cave—an ∼7 × 7 m conical, dome shaped hollow at about 1.8 m at its highest (Fig. 1, *Top* and Fig. 5, *Top Left*). Rock-art found on the ceiling is monochrome red. These elements include the Pinwheel element (Fig. 1, *Bottom Left* and *SI Appendix*, Fig. S1) and the attendant Transmorphic figure (*SI Appendix*, Fig. S2) having antennae, dichoptic eye orbits, and an elongated body with four appendages each with three fingers/toes. Other ephemeral red paintings such as a circular figure, fragments, and possible dots are also found on the cave ceiling. A sandstone asphaltum lined tablet which may have been a hopper mortar lies on the surface (Fig. 6, *Middle Left*). In the ceiling, a minimum of 56 porous hollows containing fibrous quids were identified, then mapped within the conglomerate formation (Fig. 6, *Top Left*). Eight of these 56 hollows had their contents removed, with each proving to contain from 1 to 10 quids. This resulted in the 15 quids comprising this study.

Occupational evidence was found in every excavation, among the BRMs, on the landform immediately outside the cave, at the entrance to the cave, and inside the cave (41). Burnt bone, fire-affected rocks, and high quantities of charcoal show that cooking and eating occurred on both sites. Groundstone artifacts indicate the preparation of nut and seed foods for consumption. Debitage shows that reduction processes were late-stage manufacture or retouching. Projectiles found indicate that the making and maintenance of points for hunting was a major activity at both sites.

### Paleobotanical Analysis.

Archaeobotanical samples from the BRM site and Pinwheel Cave show that a diverse array of plant foods were being processed and consumed. Nuts and fruits, including acorn (*Quercus* spp.), juniper (*Juniperus* sp.), and elderberry (*Sambucus* sp.), were identified at both sites, and small edible seeds (*SI Appendix*, Tables S3 and S4). Grass seeds were the most common seed recovered, making up 81% of the small seed assemblage at the BRM site, and 74% at Pinwheel Cave. Most of the grass seeds were identifiable to family only, but several varieties were present, including hairgrass (*Deschampsia* sp.), wild barley (*Hordeum* sp.), bluegrass (*Poa* sp.), and fescue (*Festuca* sp.). All taxa recovered occur primarily in grassland/prairie (∼60%) and oak woodland (∼31%) plant communities, but, at Pinwheel Cave, 7.8% of the taxa come from wetland plant communities.

Cattail (*Typha* sp.) and Indian rush (*Juncus* sp.) were identified at both sites, but Pinwheel Cave had higher densities, plus three additional wetland taxa: pondweed (*Potamogeton* sp.), tule (*Schoenoplectus* sp.), and alkali sacaton (*Sporobolus* sp.). Importantly, all of these wetland taxa are used in basketry/weaving. It is possible that these basketry materials were stored or dried in the cave, and/or that weaving activities occurred at Pinwheel Cave. Wild cucumber (*Marah* sp.) is one of the most ubiquitous taxa recovered in California archaeological sites. The large, oily, toxic seeds had a variety of uses (including as a pigment binder or salve), but they also ignite easily and could have been used as tinder/kindling (56). Other potential tinder taxa represented at Pinwheel Cave include cattail fluff and acorn nutshell.

No seeds or other morphologically identifiable fragments of *Datura* or other psychotropic plants (e.g., tobacco) were recovered. However, given the ethnographically reported preparation methods for *Datura*, the absence of morphologically identifiable remains is not necessarily surprising. The archaeobotanical assemblages for these sites indicate that they were both areas of subsistence-related activities. The Pinwheel Cave assemblage was more diverse (Shannon−Weaver diversity index = 1.73) compared with the BRM site (diversity index = 1.49), and had higher density of plant remains overall. While preservation may be better inside Pinwheel Cave, these patterns indicate that subsistence activities occurred at both sites, and possibly other domestic activities like basket making/preparation.

### Chronological Results.

The radiocarbon record from Pinwheel Cave and the nearby BRM site (*SI Appendix*, Table S6) indicates that these sites were occupied during the Late Period (650 B.P. to 168 B.P.; AD 1300 to AD 1782; Fig. 6, *Middle Right*). The earliest date is from quid H, which has a 95.4% calibrated range of 420 cal B.P. to 295 cal B.P. (AD 1530 to AD 1655). Two samples from TP1 in the level from 20 cm below surface (cmbs) to 30 cmbs of Pinwheel Cave have 2σ calibrated ranges that extend until ∼AD 1890 (240 cal B.P. to 60 cal B.P. and 235 cal B.P. to 60 cal B.P.; AD 1710 to AD 1890 and AD 1715 to AD 1890), the date we assigned as the terminal date of occupation of the region. There are no obvious reversals in the calibrated dates from Pinwheel Cave, indicating that the stratigraphy is likely intact. These modeled dates range from 305 cal B.P. to 140 cal B.P. (AD 1645 to AD 1810; 95.4% range; 40 cmbs to 50 cmbs) to 235 cal B.P. to 60 cal B.P. (AD 1715 to AD 1890; 95.4% range; 20 cmbs to 30 cmbs), indicating deposition over a relatively short time. This is consistent with the dates obtained from the quids from the cave ceiling. These dates range from 420 B.P. to 295 B.P. (AD 1530 to AD 1655; 95.4% range; quid H) to 270 cal B.P. to 85 cal B.P. (AD 1680 to AD 1865; 95.4% range; quid C), overlapping with the stratigraphic dates, indicating that people deposited materials on the floor of the cave at the same time quids were left in the ceiling. Because these dates fall during a well-established plateau in the radiocarbon calibration curve, the 95.4% ranges are all broken into two or more ranges, so the dates given here indicate the maximum possible scope of these ranges.

The two dates from the BRM area are also consistent with those from Pinwheel Cave with 95.4% ranges from 380 cal B.P. to 105 cal B.P. (AD 1670 to AD 1845; TP4, 60 cmbs to 70 cmbs) to 345 cal B.P. to 85 cal B.P. (AD 1605 to AD 1865; TP4, 20 cmbs to 30 cmbs). The uncalibrated date for the deeper sample is 155 y younger (855 B.P.) than from the shallower sample (1000 B.P.). There is enough overlap in the calibrated ranges to successfully create a stratigraphic model for these dates, but it is possible that there may have been a reversal in the samples from sediment mixing. The most likely scenario is that the material from this sequence was deposited relatively quickly.

At both the Pinwheel Cave and BRM sites, the bead typology confirms that occupation occurred from the Late Period to the Historic Period (*SI Appendix*, Tables S1 and S2). Glass beads and iron needle-drilled *Olivella* wall beads would have been available after the establishment of missions beginning in AD 1782. Many of the other *Olivella* wall beads had chipped edges and may thus also be indicative of Historic era production. A glass wire-wound bead and two percussion caps show that the use of the site continued well into the American Period (beginning in AD 1848). Small, triangular projectiles all indicate Late Prehistoric to Historic Periods.

## Discussion

The SEM analysis identified that 14 of the 15 quids are, in fact, *Datura*, while we were able to successfully extract and identify the hallucinogenic alkaloids atropine and scopolamine from four quids. The 3D microscope imaging revealed that the quids were highly processed, cut into individual clumps, then crushed and matted, and, in all probability, masticated. Each quid appears therefore to have been a single “dose,” inserted into the mouth and chewed/sucked in order to extract the hallucinogenic alkaloids. This likely took place in the cave, after which the quids were inserted into the cavities above. Importantly, the archaeological evidence indicates group activities in the cave. Projectile points, debitage, and the arrow shaft straightener indicate the manufacturing and/or retooling of weaponry in preparation for hunting forays. The artiodactyl and small mammal remains support that hunting activities did occur, with game brought back and consumed on site and in the cave. Groundstone and paleobotanical evidence shows that seeds and other plant materials were gathered for food processing, consumption, and possible storage. The ingestion of the *Datura* therefore did not preclude the use of the site for other activities, showing that the site was not one of shamanic exclusion. However, because fasting is almost universally part of the *Datura* ritual, food was not likely to have been consumed during the ritual, and likely represents the differential use of the site seasonally as a temporary residential base.

While 56 clearly identifiable quids have been located in the crevices, this is only a partial representation of the total number that were once extant, as small remnants of fibrous materials across the ceiling indicate that quids may have previously been inserted into crevices but are now gone. Within the deposits in the cave, periodic water action may limit the survival of organic material, especially in the lower levels, but some fibrous fragments were noted during excavation in the upper portions of the excavation units. The majority of the interior deposits of the cave have not been excavated, so the extent of organic survival remains unknown. Importantly, some of the crevices contain multiple quids. Three of the eight hollows which we sampled (F, G, and H; *SI Appendix*, Fig. S4) contained pairs inserted together, while sample 2 contained 10 quids. It is within this packet that we were able to identify atropine and scopolamine. Given the toxicity of the plant, the best explanation is that this packet represents the doses of a group, perhaps up to 10 individuals, matching what would be expected from a group initiation ceremony. However, ethnographic information always associates adolescent group initiations with drinking toloache, not chewing *Datura* directly. It is likely that that this site represents an undocumented means of initiation, but it may be that the chewing of Datura could have been related to other group ceremonies such as preparation for hunting expeditions. The chewing of *Datura* could have also occurred for an individual to obtain personal power or for using *Datura* for medicinal purposes.

Given the evidence for *Datura* preparation and consumption, it is now possible to interpret the rock art based upon associated evidence. Both the reflectance transformation imaging (RTI) and portable X-ray fluorescence analyses show that the Pinwheel painting had different phases and pigment applications. It appears that an earlier image was painted over by the Pinwheel, which, in turn, was retouched several times. The painting is therefore not the product of a single painting event, and therefore probably is not a depiction of a singular artist’s vision. Rather than representing entoptic phenomenon of the ASC model, the large Pinwheel image is almost certainly a representation of the actual *Datura* flower. This large pinwheel motif specifically has the characteristics of the opening of the bud into its large white flower. As the *Datura* bud opens, the carolla unfurls into five arms, each curving back to create a pinwheeling appearance. Opening at dusk, it attracts crepuscular pollinators in the family Sphingidae, commonly referred to as “sphinx,” “hummingbird,” or “hawk” moths. Multiple species of hawkmoths are found in the landscape, with the most common being the white-lined sphinx moth, *Hyles lineata* (Fabricus) (*SI Appendix*, Fig. S1, *Lower Right*). Around sundown, this species is found hovering at the entrance of *Datura* flowers, providing pollination services while inserting a long proboscis to the flower’s base to drink nectar. With the antennae, dichoptic eye orbits, and tapering abdomen, the head and body of the attendant Transmorph appears to represent some form of insect, perhaps representing the hawkmoth. Botanists have observed hawkmoths behaving erratically as if intoxicated after pollinating *D. wrightii* (57). With four appendages, the Transmorph appears to be anthropomorphized. So, just as the hawkmoth consumes nectar from the *Datura* flower and exhibits its effects, the Transmorph may represent the *Datura* taker who will exhibit analogous behavior. While the discovery of the preparation and consumption of *Datura* at Pinwheel Cave is unique, this *Datura*/hawkmoth symbolism is not uncommon in other areas of the American West (32, 36, 37). However, the iconography at Pinwheel Cave is likely to be an independent local development logically arising from regional artistic traditions and through close and direct observations of the distinctive ecology of the *Datura* plant. This would have been understood within Native Californian mythological and ontological foundations (12, 13). The art is thus crucial as a visual accompaniment to the setting within which the consumption of the plant takes place. This has significance in the wider ASC debate concerning the role of trance in the origination of rock art. Rather than the art depicting what is seen in trance, the Pinwheel instead is likely a representation of the plant causing the trance. Instead of a shamanic self-depiction, the Transmorph may represent an insect such as the hawkmoth, who consumes nectar from the *Datura* flower before coming under the influence of its effects, thus exhibiting behavior analogous to those consuming *Datura* in the cave. The rock art served epistemologically, preparing participants for the experience they were undertaking by inculcating them into culturally specific knowledge concerning the plant and its effects. The rock art thus established the space where individuals underwent a deeply meaningful first-hand entheogenic experience within the context of an important communal site. If true, then the art codified important constituent elements of the entheogen which were then visible to the participants, but also to the wider community who utilized the site for other purposes such as hunting, gathering, tool maintenance/manufacturing, food preparation, and consumption. Thus, while establishing unambiguous evidence for the consumption of a hallucinogenic substance at a known rock art site, this study calls into question assumptions that rock art imagery directly reflects private images seen in trance rather than acting as visual catalysts for communal experiences. This calls for a reappraisal of the ASC shamanic model: Rather than derivative of a shaman’s experience, rock art here, instead, is an active accompaniment to the trance event, communicating key information about the constituent elements of such events within communal contexts.

While it is clear that the quids were pounded and/or masticated, we cannot determine whether they were initially steeped in water or boiled to make toloache. The reuse of quids is documented in one Chumash oral narrative where infants sucked on used yucca quids to extract residual nutrients (18). Indeed, the lone yucca quid in our samples questions what constitutes the remaining in situ quids. Likewise, it may be that other analytical methods may be employed to identify alkaloids in the samples (58, 59). Importantly, the dating indicates that *Datura* use within the cave occurred over several generations, spanning the Late Prehistoric Period into the Historical Period. The dates establish a prehistoric context for *Datura* usage in California, showing that it did not originate in the Historical Period as a reaction to colonial events (60). Even so, the dating evidence and the lack of depth in the excavated units (*SI Appendix*, Fig. S8) present no evidence for earlier prehistoric usage at this particular site. Clearly, there is significant usage of the site in postcontact periods, including Spanish and Mexican into the American Periods (61). This continuation of *Datura* use shows the resilience of Native practice in the use of an entheogen through successive colonial regimes.

## Materials and Methods

### The 3D Digital Microscopy.

The 3D digital microscopy assessed patterns of variation expressed in quid surfaces. A Keyence VHX-2000 digital microscope with VH-Z20 20-200 magnification lens was used for microscopic imaging of the quids, and to resolve and measure features at the micrometer level. The Keyence VHX-2000 records multiple calibrated images of an object and creates a fully focused 3D model using an image stitching algorithm. Images were taken using the fine depth composite function, with the high dynamic range enabled and image pitch set to 50. All other settings remained on default.

The 3D imaging of each quid was made at 20× magnification. Each surface was arbitrarily defined as either dorsal or ventral. Measurement of depressions on both the dorsal and ventral surfaces was carried out using the Keyence’s 3D calibrated measurement function. Maximum depression depth (MDD), maximum long-axis depression diameter (MLAD), and maximum short-axis depression diameter (MSAD) were all measured on areas of interest on both dorsal and ventral surfaces. Diameters were recorded in microns and converted to millimeters (*SI Appendix*). Basic descriptive statistics for each measurement were undertaken, and a one-way ANOVA between dorsal and ventral surfaces was conducted using IBM SPSS v20. Levene’s test for homogeneity of variance was applied.

The 3D models were converted into 2D and 3D heat maps which graphically displayed the depression topography of each surface (Figs. 2 and 3 and *SI Appendix*, Fig. S4). Fit line height cross-sections were taken orthogonally across the midway long axis, and the resulting profiles were subjected to GMM of shape statistics in order to quantify and understand the variation occurring on the surface of each specimen. Simply defined, GM is the statistical analysis of form based on Cartesian landmark coordinates (62).

In this analysis, the dorsal and ventral quid surface profiles were saved in jpeg format. Each profile was loaded into TPSDig2, and 20 semilandmarks were applied to each profile curve, and then resampled within TPSDig2; landmarks were allowed to slide on the curve such that they were equidistant from each other when scaled against total length. Resulting X-Y coordinates were plotted and saved in the same order for each profile. Landmark configurations were subjected to GPA using the MorphoJ package. Following GPA, the landmarks were subjected to PCA to reduce dimensionality and investigate patterns of population variation. All imaging is available via at the University of Central Lancashire repository (45).

### Alkaloid Extraction Methods.

*Datura* leaf material and quids were extracted following the same procedure, based upon that of Bo et al. (52). Comparison of extraction methods included solid phase extraction and liquid extraction utilizing methanol or a methanol:water mixture. Results revealed the greatest atropine and scopolamine recoveries from spiked eggplant leaf’s was on average 2% greater through methanol only extraction, in comparison to the methanol:water solvent system. As such, methanol was chosen as the extraction solvent within this study. It should however be noted that alternative extraction procedures have since been published, including that of Cirlini et al. (58), which have identified that an acidified (3:2 vol/vol) methanol:water system with a small portion of acetonitrile (0.2%) provided the optimum extraction of the alkaloids from food material and as such should be considered for future plant extractions. *Datura* extractions were performed in triplicate on air-dried leaf material. To ∼50 mg of the dried plant material 10 mL of methanol and 1 mL of the internal standard mexiletine hydrochloride at a concentration of 0.15 mg mL^−1^ was added. Extractions were performed via ultrasonication at room temperature for 40 min. Preconcentration was achieved by evaporation of the extract to a final volume of 3 mL For LC-MS analysis a 1 mL portion of the extract was passed through a 0.2 µm PTFE filter to remove any solid particulates.

Quids were extracted following the same procedure as described above. To maximize the concentration of atropine or scopolamine 100 mg of each quid was extracted. The number of replicate extractions performed for each quid was dictated by the subsample size available. Subsamples eight and ten were not extracted, subsamples five and nine were extracted once. The remaining samples (two and four) were extracted in duplicate.

### LC-MS Analysis.

Extracts were analyzed by liquid-chromatography mass spectrometry (LC-MS), with positive identifications of both tropane alkaloids made based upon their pseudomolecular ion within their mass spectra. All quid extracts and standards (0.010–1.50 mg mL^−1^) were analyzed via LC-MS using an Agilent 1200 series LC-MSD with a fixed variable wavelength detector set at 214 nm, coupled to an Agilent 6130 quadrupole mass spectrometer with electrospray and atmospheric-pressure chemical ionization. An Agilent poroshell 120 Å EC-C-18, 4.5 × 7.5 mm column for reverse phase chromatography was used at a column temperature of 40 °C. Five microliters of the extracts or standards were injected with a flow rate of 1 mL min^−1^ and a total run time of 18 min. Analytes were eluted using a gradient flow profile of 0.1%vol/vol formic acid and water (mobile phase A) and 0.1%vol/vol formic acid and acetonitrile (mobile phase B). The flow profile is detailed in Fig. 4, bottom table. Results are expressed purely in a qualitative manner. Atropine and scopolamine in extracted quids were identified via comparison of retention time and detected masses to the known standards and confirmed through the use of the NIST database.

Standards were analyzed alongside quid extracts as system suitability tests. Standards contained atropine, scopolamine and mexiletine with methanol as the diluent. For all standards the mexiletine concentration was kept constant at 0.05 mg mL^−1^. All reagents were of analytical grade quality and used as received. The pseudomolecular ion of each analyte (290 *m/z* atropine and 304 *m/z* scopolamine) was used to construct the EIC to allow for identification of the tropane alkaloids within the quid and plant material.

### SEM Methods.

Examination of the 15 quids and comparative reference specimens (prepared and mounted using the same method) was undertaken in a variable pressure SEM (VP SEM), Hitachi S-3700N, using the backscatter electron (BSE) detector at 15 or 16 kV, with the SEM chamber partially evacuated (30 Pa) in order to suppress charging. Magnifications ranged from ×20 to ×400. The preferred working distance was 13.6 mm, but was raised to 18.2 mm (as required). With the BSE detector, 3D mode (rather than Compositional) was preferentially selected to maximize the opportunity to reveal diagnostic features for identification (63).

All of the comparative plant samples came from mature plants found locally on the Wind Wolves Preserve and within 5 miles of Pinwheel Cave. Samples included a range of what was considered possible or likely quid material including Jimson weed (*D. wrightii*), Tule (*Schoeneoplectus californicus*), Tobacco (*Nicotiana quadrivalvis*), Chuchupate (*Lomatium californicum*) and Yucca (*Yucca whipplei).* The samples included pieces of leaf, stem, and subsurface rootlets. A complete inventory SEM images of each comparative sample is available at the University of Central Lancashire repository (45).

### Paleoethnobotanical Methods.

Bulk soil samples (1 L each) were collected from each excavation unit at arbitrary 10-cm levels. Samples were processed with tap water using the Flote-Tech machine-assisted flotation system at University of California, Santa Barbara (UC Santa Barbara). Light fraction materials of >0.5 mm, and heavy fraction materials of >1.0 mm, were examined for carbonized macrobotanical remains. Each light fraction sample was size sorted (>2.0 mm, >1.4 mm, and pan) and analyzed under a stereomicroscope. Only carbonized remains were considered in the analysis, due to the presence of rodent activity and fecal matter. All of the carbonized material from the 2.0-mm-size grade was quantified, including seeds, nutshell, and wood charcoal. No wood charcoal was quantified from smaller mesh sizes, and no wood identification was conducted. Acorn nutshell, which tends to break into small pieces, was quantified from all sizes, as was wild cucumber. Three offsite auger samples were analyzed to confirm that the charred remains recovered from midden areas were cultural versus natural in origin. Identifications were conducted through use of the comparative collection at UC Santa Barbara’s Integrative Subsistence Laboratory and the Seed Identification Manual (64, 65).

### Absolute Dating Methods.

Radiocarbon measurements were calibrated in three groups to obtain 1) the chronology of occupation for the stratigraphic context of Pinwheel Cave, 2) the stratigraphic context associated with the nearby BRM, and 3) the quids recovered from the ceiling of the cave. For categories 1 and 2, a priori information about the relative chronology of samples allowed us to develop stratigraphic models to refine 95.4% calibrated radiocarbon ranges. For category 3, no similar information was available. We calibrated all of the dates in OxCal 4.1 using the most recent terrestrial (IntCal13) and marine (Marine13) calibration curves. For *Olivella biplicata* samples, we used a variable ∆R value for the Santa Barbara Channel region. We used the sequence command in OxCal to further constrain the error ranges on dates based on the relative stratigraphic position of the radiocarbon samples within each of the units. The two dates from TP1, 20 cmbs to 30 cmbs from Pinwheel Cave, were modeled as a phase within the sequence, because their relative deposition pattern was unknown and they might have been contemporaneous. We used the before command to constrain the calibrated ranges to predate 1890, the putative latest date for Native access to the site.

## Supplementary Material

Supplementary File

Supplementary File

## Data Availability

All data needed to evaluate the conclusions in the paper are present in the paper and/or *SI Appendix*. In addition, 3D imaging, RTIs, and SEM data are accessible at UCLanData, https://doi.org/10.17030/uclan.data.00000213 (45). All data underpinning the chromatographic work are openly available from the University of Strathclyde KnowledgeBase at https://doi.org/10.15129/6d5b0425-1204-4b4f-b33d-201774c45bbc (66). We also provide RTIs online using the Visual Media Service to facilitate easy access. To access the files please visit Pinwheel pictograph, http://visual.ariadne-infrastructure.eu/rti/pinwheel-pictograph; DStretch Pinwheel pictograph, http://visual.ariadne-infrastructure.eu/rti/dstretch-pinwheel-pictograph; Transmorph Pictograph, http://visual.ariadne-infrastructure.eu/rti/transmorph-pictograph; and DStretch Transmorph pictograph, http://visual.ariadne-infrastructure.eu/rti/dstretch-transmorph-pictograph.
