# Supplementary material for: Datura quids at Pinwheel Cave, California, provide unambiguous confirmation of the ingestion of hallucinogens at a rock art site
Source: Proc Natl Acad Sci U S A. 2020 Nov 23;117(49):31026–37. doi: 10.1073/pnas.2014529117 (PMC7733795; doi:10.1073/pnas.2014529117)
Supplement: Supplementary File [file pnas.2014529117.sapp.pdf]

PNAS
www.pnas.org

Supplementary Information for

Datura quids at Pinwheel Cave, California provide unambiguous confirmation of the ingestion of hallucinogens at a rock art site.

David W. Robinson,[1]* Kelly Brown,[2] Moira McMenemy,[2] Lynn Dennany,[2] Matthew J. Baker,[2] Pamela Allan,[2] Caroline Cartwright,[3] Julienne Bernard,[4] Fraser Sturt,[5] Elena Kotoula,[6] Christopher Jazwa,[7] Kristina M. Gill,[8] Patrick Randolph-Quinney,[9] Thomas Ash,[1] Clare Bedford,[1] Devlin Gandy,[10] Matthew Armstrong, [11] James Miles, [12] and David Haviland [13]

1. School of Forensic and Applied Sciences, University of Central Lancashire, Preston, PR1 2HE, United Kingdom.
2. WestCHEM, Department of Pure and Applied Chemistry, University of Strathclyde, Glasgow, G1 1RD, United Kingdom.
3. Department of Scientific Research, British Museum, London, WC1B 3DG, United Kingdom.
4. Department of Anthropology, Geography, and Geology, East Los Angeles Community College, Monterey Park, 91754, United States of America.
5. Department of Archaeology, University of Southampton, Southampton, SO17 1BJ, United Kingdom.
6. Information Services Group, University of Edinburgh, Edinburgh, EH8 9YL, United Kingdom.
7. Human Paleoecology and Archaeometry Laboratory, Department of Anthropology, University of Nevada, Reno, 89557-0096, United States of America.
8. Museum of Natural and Cultural History, University of Oregon, 97403, United States of America.
9. Department of Applied Sciences, Faculty of Health and Life Sciences, Northumbria University, Newcastle Upon-Tyne, NE1 8ST, UK; and Department of Human Anatomy and Physiology, Faculty of Health Sciences, University of Johannesburg, PO Box 524, Aukland Park 2006, Johannesburg, South Africa.
10. Department of Archaeology, University of Cambridge, CB2 3DZ, United Kingdom.
11. Pacific Gas and Electric Company, United States of America
12. Division of Cathedral and Church Buildings. Church of England, Southampton, SO17 2FY, United Kingdom.
13. University of California Cooperative Extension, Kern County, Bakersfield, California, 93307, United States of America.

David Robinson
Email: dwrobinson@uclan.ac.uk

www.pnas.org/cgi/doi/10.1073/pnas.2014529117

This PDF file includes:

Supplementary text
Figures S1 to S8
Tables S1 to S6
Legends for Movies S1
Legends for Datasets S1
SI References

Other supplementary materials for this manuscript include the following:

Movie S1

Supplementary Information Text

Reflective Transformation Imaging results*:* Reflectance Transformation Imaging (RTI) is a virtual relighting technique used for surface topography analysis, derived from a sequence of raking light images. Although RTI has been largely applied for the study of petroglyphs (1) it proved to be useful for the study of painted surfaces (2, 3). In combination with Decorrelation Stretch (4), common digital image enhancement technique for faded pictorial elements, RTI can provide insights for pictographs (3). RTI was applied to the Pinwheel and Transmorph designs (see **Fig. S1, S2**). After the acquisition. datasets were processed with RTIBuilder using the hemispherical harmonics (HSH) fitting algorithm. Using the DStretch plugin and Image J batch processing tools, DS RTI dataset was generated and then processed following the mainstream methodology, resulting in DS RTI files. The RTI files were viewed in RTIViewer.
Imaging work was undertaken to determine if the pictographs were single compositions or made in multiple phases. The Pinwheel design is more complicated than it appears in conventional photography. Differences were identified in five areas (**Fig. S1, upper left**) and remnants of another layer indicate later additions (**Fig. S1**). Near the Pinwheel, is a painting known as a Transmorph: variation in colour and texture across it is due to changes in the substrate rather than different pigments (**Fig. S1, lower left**). Close up imaging of the Transmorph clarify the form of the dichoptic eye orbits, extending 'cheeks', and antennae (**Fig. S1 lower left and center**). An exfoliated portion of the rock surface truncates the left antennae (from the viewer's perspective) so that it originally was longer. The right antennae has the mid-portion missing due to exfoliation, but can be seen bending outward in the dStretch enhancement (**Figure S1, bottom center**).
Portable X-ray Florescence**:** Analysis of pigments used a Bruker Tracer III handheld X-Ray fluorescence spectrometer set at 40kV and 3.4uA. S1PXRF software accumulated each spectrum over 60 seconds. The analysis compared the relative number of counts per second of particular elements by using ARTAX software to calculate the net area under each elemental peak and converting in total counts examined using Microsoft Excel and PAST statistical packages. All discussion of compositional data refers to a proportion of counts presented as a percentage of total counts detected by the instrument. These include principal component analysis (PCA) using a covariance matrix and a Mann-Whitney test to identify any significant differences between areas of pigment. For the Pinwheel, total 49 spectra were accumulated, 16 from the substrate and 33 from the pigment from points shown in **Fig. S1**, **upper right**. Readings were taken to determine if different pigments were utilized. The substrate around the Pinwheel has a higher proportion of calcium counts than the paint and is compositionally consistent except for two outlying assay points. The PCA plot (**Fig. S1, Middle right**) shows the clustering and variation of substrate and pigment points. Spectra from pigment assays shows silicon, potassium, iron and arsenic at higher relative abundances than found in the substrate (**Fig. S1, Middle right**). The elements with the highest proportion of counts in pigment spectra are iron, potassium and silicon, and PCA analysis shows that the key elements explaining variation between pXRF assays are iron, calcium and arsenic.

The abundance of iron within the spectra, along with the detection of silicon, indicates an iron oxide based pigment such as ochre was most likely used to create the painting. Arsenic can occur naturally as a component of red ochre at some source locations (5). The proportion of arsenic counts (**Fig. S1, Middle right**) varies from 0.28% to 1.87% of total counts within the Pinwheel but this variation in the relative proportion or arsenic counts correlates with variation in iron counts within the spectra and could be explained by variation in thickness of a single pigment material. However, if variance in the pXRF data is solely explained by covariance in iron and arsenic in a single pigment applied in areas of varying thickness, the ratio of iron and arsenic should be consistent throughout the pigment assays. **Fig. S1, Middle right** shows the correlation between iron and arsenic pXRF counts. The points circled in this plot are a group which do not appear to fit the correlation observed in the other points. To test the consistency of this correlation the ratio of the proportion of arsenic and iron counts was calculated for each assay and these ratios (**Fig. S1, Middle right**).

The points indicate that there is a difference between the pigment material in the main part of the motif and the smaller group containing points 1, 5, 6, 16, 17, 18, 19, 28 and 31. The ratios of As/Fe for each group of assay point were entered into a Mann-Whitney test to determine if the two groups are significantly different. The nul hypothesis was that the means are the same and the p-value produced was 0.0000136, indicating a significant difference between the two groups based on their iron to arsenic ratios. This indicates that two different pigments may have been used in the Pinwheel, a result consistant with the RTI analysis above.

28 pXRF spectra including 22 from the pigment and 6 from the substrate were accumulated from the transmorphic figure at Pinwheel (**Fig. S3**). Similar to the pinwheel motif arsenic counts were identified in some spectra from the anthropomorph. However, although spectra from the body of the anthropomorph show clearly elevated arsenic counts relative to the substrate, only a very small difference is seen between the substrate arsenic count and that of the limbs (see **Fig. S3**). This implies that only one pigment was used in the making of the transmorph.

LC-MS Analysis of Datura Plant Material: Extraction of atropine and scopolamine from *Datura* plant material ensured the tropane alkaloids could be removed from the complex sample matrix. Extractions were performed via ultra-sonication, with each extract pre-concentrated and filtered to remove any solid particulates. Analysis was performed using the same chromatographic method as the standards. The extracts of the plant material showed the presence of both atropine and scopolamine. Neither alkaloid could be detected within the UV chromatogram as concentrations present were below the limit of detection of the chromatographic analysis. Extracted ion chromatograms (EIC) were used to identify the presence of the alkaloids. The EIC were created using the mass of pseudo-molecular ion for each alkaloid, 290 m/z for atropine and 304 m/z for scopolamine. This allowed for the presence of the analytes to be determined with ease. Their low concentrations and the large number of species co-extracted with the tropane alkaloids of interest prevented their identification within the total ion chromatogram (TIC). The chromatographic analysis and EIC of the plant material can be seen in **Fig. S5** and **Fig. S6**.

Archaeology, mapping, and quid sampling: Located in Kern County, South-Central California (**Fig. 1, top left inset**) the complex of Pinwheel Cave and its associated bedrock mortar complex (CA-KER-5836, 5837) covers an area approximately 380 x120 meters (see **Fig. S7, upper left**). The locale is characterized by open oak woodland on ridgelines and north facing slopes. On the northwest margin of the locale, nine bedrock mortar (i.e. BRM) stations with 19 BRMs (CA-KER-5837) (**Fig S7, lower left and right**) is a common regional food processing station for the pounding of acorns and other nuts and seeds. Towards the south from the BRMs, a large conglomerate formation can be spotted amongst oak trees (see **Fig. S7, upper right**). This large formation contains Pinwheel Cave (CA-KER-5836). An augur transect running from the BRM complex to the cave site found no evidence of occupation on the land-forms between the sites (**Fig. S7 upper left**).

Archaeological excavations focused inside the cave and immediately outside the cave, plus at a nearby bedrock mortar (i.e. food processing) complex (**Fig. S7**). Six test-pits were excavated at the bedrock mortar complex, while three test-pits focused within the cave and one outside (**Fig. S8)**. All units were excavated in 10 cm spits, with all deposits sieved in 1/8th inch screens.

As part of the 2007 project an extended topographic survey was carried out to help create a digital terrain and geomorphological model. During this survey the absolute accuracy of the UTM coordinates referred to in this study was ascertained. A handheld GPS unit accurate to within 8m in three-dimensions was utilized, with heights noted above ellipsoid rather than geoid. As such, all co-ordinates obtained from total station work and used in this study are internally accurate to within millimeters, but strictly geolocated within an 8m total tolerance. The interior of the cave was also mapped using a Topcon Total station, including the location of the artwork and each of the quids on the ceiling. We also utilised a Faro Focus 3D, a small lightweight scanner that was utilized to create a 3D point cloud of the interior and exterior of the rock formation. 58 scans, each including 80–90 photographs were taken inside at different positions spaced at roughly one metre intervals. Three stationary reference spheres were placed in order to reference the scans together. Using reference spheres within each scan these were stitched together using Scene software to produce a point cloud. This was then exported as a PTS point cloud into Cloud Compare, subsampled to reduce the file size and exported as a PLY file. Pointools was then used to render and animate the 3D model (**Movie S1**).

A total of eight of the packed fibrous quids found in natural pockets within the ceiling of the cave were sampled. A pair of needle-nose pliers was used to extract the quids: this implement was sterilized by boiling in water. Surgical gloves were worn during extraction, with samples pried from the ceiling directly into foil-lined plastic boxes. Thus the samples were not handled or touched during sampling. In 2007 two initial samples were taken, Quid 1 and Quid 2. Quid 1 was a dark brown fibrous example taken from the ceiling immediately within the eastern entrance to the cave. Quid 2 was a large tan pulpy example towards the northern entrance. In the lab, it was later discovered that Quid 2 was in fact a packet of ten individual quids tightly packed together within the crevice. Each of these quids were then label Subsample 1, Subsample 2, and so forth. Note that two Subsamples were not utilized as part of this study. In 2016, eight more samples labeled A through H were identified. However, only six samples were removed from the crevices – B, C, E, F, G, H (A and D remain in situ).

Beads from the Pinwheel Cave Site (Ker-5836): Excavation of the 4 test pits at the Pinwheel Cave site produced a total of 25 beads (**Table S1**). Both shell and glass beads were recovered, but although this site contained a wider variety of bead (and shell material) types than Ker-5837, it did not contain all the same bead types present there (such as stone beads or *Olivella* spire-ground beads). Most of the Pinwheel Cave beads are made of shell (n=21). *Olivella* beads are the most abundant (n=15), making up half of the assemblage. *Olivella* wall beads are the most common type (n=8), followed by lipped beads (n=3), and cupped beads (n=2). Two additional *Olivella* wall beads appear to have been perforated using iron needles. Beads made from the *Mytilus* shell were relatively well represented for such a small sample, with both disc (n=3) and cylinder (n=1) beads making up 16% of the assemblage. *Haliotis rufescens* disc beads were also present, although in small numbers (n=2). Glass beads (n=4) were also recovered from Ker-5836. Two of these are blue drawn, monochrome, undecorated beads, which are common for Historic sites in California. Another glass bead recovered appears also to be a drawn bead but has been badly burned such that color and a detailed type could not be assigned with confidence. The fourth glass bead is an uncommon black, decorated, spheroidal, freewound glass bead. Beads were recovered from Test Pits 1, 3, and 4. No beads were found in Test Pit 2, but it is possible that this is an outcome of its smaller size (0.5 x 1m). Most of the beads were recovered from Test Pit 1 (n=11) and Test Pit 3 (n=9), with fewer beads found in Test Pit 4 (n=5). In Test Pits 1 and 3, located within the cave and at its entrance, respectively, all but one bead were recovered from the upper 40 cm. The largest number of beads occurred in the 30-40 cm level, which appears to be the base of the most significant occupational level for the cave. This level also shows the most diverse range of bead types, containing every type of bead present at the site except *Haliotis*. Although stratigraphic mixing is always a possible complicating factor, the presence of needle-drilled and glass beads suggests that Historic-era occupation of Pinwheel Cave began at the 30-40 cm level. The spheroidal, freewound glass bead was recovered from the 10-20 cm level of Test Pit 3, suggesting that use of the cave continued (at least sporadically) until the Mexican or perhaps early American period (1820s-1850s). Test Pit 4, located outside the cave, appears to have a unique depositional pattern. There, cultural materials are most abundant

in the 30-40 cm level and deeper, but it is unclear if this is the result of earlier and longer-term use of this area of the site or if it is the outcome of variable depositional processes. The lack of beads above the 30-40 cm level and the presence of beads as deep as 60-70 cm there does not necessarily indicate significantly earlier occupation. However, since no unambiguously Historic bead types (i.e., needle-drilled or glass beads) were recovered from Test Pit 4, it is possible that it reflects an earlier phase of occupation (i.e., during the Late period). It is interesting, however, that Test Pit 4 contains the only *Olivella* lipped beads (n=3) present at the site. Interpretations of this pattern will be discussed below.

Beads from the Pinwheel BRM Complex (Ker-5837): Fewer beads were recovered from the Pinwheel BRM Complex (Ker-5837) than the Pinwheel cave site (Ker-5836) (**Tables S1** and **S2**). This is not unexpected, as the Ker-5837 test pits yielded fewer cultural materials overall. In total, 14 beads were recovered from Ker-5837 (**Table S2**). While many bead types were present at both sites, the assemblage from Ker-5837 does not contain beads made from *Haliotis rufescens* or *Mytilus californianus*, but does contain a stone bead and *Olivella* spire-ground beads, types missing from Ker-5836.

Shell beads were the most abundant. Many of the shell beads recovered from Ker-5837 show signs of extensive weathering from exposure to sunlight and moisture. *Olivella* wall beads (n=5) are the most common bead type, making up 36% of the assemblage, and 2 additional wall beads appear to have been drilled using iron-needles. *Olivella* lipped (n=1), cupped (n=1), and spire-ground (n=2) beads were also recovered. In addition to the beads, one fragment of *Tivela stultorum* (Pismo clam) detritus was recovered from Ker-5837 (**Table S2**). It cannot be determined whether this specimen is a fragment of a *Tivela* bead, a comparatively rare and valuable bead type, or whether it reflects some degree of shell modification at the site. The two glass beads in the assemblage are both drawn, monochrome, and undecorated, and they are of colors (blue and green, respectively) that are common at other Historic era sites. The stone bead recovered was made from a golden brown-black schistose material resembling muscovite schist.

Beads were recovered from four of the five test pits excavated at Ker-5837 (**Table S2**). No beads were recovered from Test Pit 5. Most of the beads found at this site occurred in Test Pit 4 (n=10), while Test Pit 1 (n=1), Test Pit 2 (n=2), and Test Pit 3 (n=2) contained notably smaller quantities. This may reflect more intensive use of the Test Pit 4 area of the site, but may instead have resulted from an atypical episode of bead deposition. All but two of the beads were recovered from the upper 30 cm of the site, and based on the presence of glass beads and needle-drilled shell beads, it appears that the majority of activity at the site occurred during the early Historic period. The two beads recovered from lower levels were *Olivella* spire-ground beads, the only bead type present that may (although not necessarily) have been deposited earlier than the Late period.

Faunal analysis details: An initial sort of the cultural materials from the Pinwheel sites was performed shortly after excavation, separating the faunal remains from other cultural and non-cultural materials. The faunal remains were brought to Santa Cruz, California, where they were further sorted by the author into narrower categories and basic analysis was performed. Most bone was in the form of small fragments that lacked all identifiable markers other than bone size or cortical bone thickness. Based on visible characteristics, all faunal remains were placed into one of the following categories:

- Small Fauna – animals that are of rabbit size and smaller
- Sm-Med Fauna – animals approximately the size of a skunk, or possibly juvenile medium Fauna
- Medium Fauna – animals approximately the size of a dog
- Med-Lg. Fauna – animals at least the size of a dog and smaller than a deer
- Large Fauna – animals the size of a deer or larger
- Bird
- Reptile/Amphibian Vert – only the vertebrae were clearly identifiable, indicating that other bones are mixed in with small fauna
- Fish
- Burrowing Rodents – primarily the head elements of gophers, ground squirrels, and the like.

- Undifferentiated – Bone that could not be classified
- Shell

Fragments of bone that retained identifying markers such as articular ends or distinctive bone morphology were taken to the faunal labs at UC Santa Cruz for further identification. These bones were compared to bones in the comparative collection, and where a match could be made, were classified to the appropriate genus or species. Nine of these bones could not be identified, but might still be identifiable yet given a larger comparative collection. Because of the small number of identifiable elements and the fragmentary nature of the bones (for example, both distal and proximal ends of a deer long bone were found, but in different units), measures such as MNI and NISP are not very informative. Such measures are of limited utility when dealing with such fragmentary collections, and to rely on these measures may lead to misleading results. Should a larger number of bones eventually be collected from this site, these measures might prove more useful.

After bone had been sorted into the appropriate categories, it was counted and weighed on a scale with 0.1 gram accuracy. Some of the bone samples were small enough that they did not register on the scale. Initially these samples were assigned a nominal weight simply to better compare them to the other bone samples, but no suitable nominal weight could be developed and this approach provided results that did not appear to provide useful information.

Faunal details from Pinwheel Cave KER-5836: A total of 1046 bone fragments weighing in excess of 405 grams were collected from four units at this site resulting in 2 cubic meters of soil excavated, for an average of 525.5 bone fragments per cubic meter of excavated soil. The majority of the bone was recovered from units 3 and 4, located at the cave mouth and outside of the cave, which account for 39% and 29% of the bone fragments recovered, respectively. The two units within the cave combined account for 32% of the bone fragments recovered. This would appear to indicate that the bulk of bone deposition occurred either at the mouth of the cave or outside of the cave. However, if the density of bone per cubic meter of soil excavated is considered, then the mouth of the cave remains the most important bone deposition area, but the interior of the cave becomes a more important bone deposition area than the exterior. The larger amount of bone at the mouth of the cave may have been due to the fact that the entrance would likely be the busiest part of the cave, as it was used for both entrance and exit, and as the limited room within the cave might result in those not immediately involved in activities within having to wait or observe at the mouth.

However, it should be noted that the topography of the site is such that sediments appear to be routinely transported both to the cave mouth and into the cave and as a natural as well as cultural bottleneck, the mouth of the cave would be likely to accumulate both cultural and non-cultural materials.

The largest category of bone is large fauna (32.41% of the bone by count, 90.56% by weight), followed by undifferentiated bone (28.39% by count, 2.82% by weight) and small fauna (23.52% by count, 3.58% by weight). Identifiable burrowing rodent bone comprises 4.3% of the bone count and 0.54% of the bone weight, and at least a portion of the small fauna is likely also from burrowing rodents. Reptile and amphibian bone comprise 1.53% of the bone count and 0.3% of the weight, and a portion of the small fauna is likely also fragments of reptile/amphibian bone (**Table S5**).

The aquatic fauna collection, composed of fish bone and shell, provides approximately 7.75% of the total faunal count, but less than 1% of the total weight. Given the relatively high shell-to-meat weigh ratio for shellfish, these appear to have contributed only a small amount to the overall diet. However, their presence is important, as shell and fish can be taken by those unable to participate in the hunt for larger game, and this may be indicative of meat contributions by a larger proportion of the populace. Unfortunately, the fish bone could not be identified to species or genus with the available comparative collections, and the shell was so fragmentary that other than noting that the majority of it came from some form of clam, it could not be identified to species or genus.

Of the large fauna, 33.92% showed signs of burning (at least 25% of the surface blackened from heat), and 15.04% was calcined to some degree. Of the small fauna, 10.98% had been burned to some degree, and 2.44% was calcined to some degree. Of the medium fauna, 21.55%

exhibited signs of burning, and 6.06% was calcined to some degree. Given that the large fauna bone is likely primarily from animals that had been hunted for food, that a larger proportion of that bone should show signs of burning than the small fauna is consistent with the interpretation that the small fauna collection is comprised of a mix of intrusive bone and bone from food animals. Likewise, it is unsurprising that the proportion of undifferentiated fauna bone burned is between that of large fauna and small fauna. Unfortunately, the other categories of bone are composed of such small samples that it is not possible to derive any meaningful information regarding the proportion of burned vs. unburned bone.

Three pieces of large fauna bone have cut marks, two others have marks that may be due to butchering. Other bones contain marks that superficially look like they may be from butchery, but on closer examination appear to be from either natural causes or from excavation tools.

A total of 20 bone fragments were taken from the collection for KER-5836 for more precise identification. These included eight bone fragments from deer (a tibia fragment, a carpal fragment, three humerus fragments, a femur fragment, an ulna fragment, and a scapula fragment), four squirrel bones (two right femurs, one left pelvis, and one left humerus), two pocket gopher elements (a right tibia and a left humerus), and Jackrabbit (one left pelvis). Other bone that could not be identified to genus or species, but that could be identified beyond the categories mentioned above include the distal end of a scapula from a large animal (possibly a large canine), one ulna fragment came from a burrowing rodent, and two rib fragments came from an animal in the size range of a deer, but lacked clear diagnostic features.

Faunal details from Bedrock Mortars KER-5837: A total of 304 bone fragments weighing in excess of 75 grams were collected from five units at KER-5837, resulting in 3.3 cubic meters of excavated soil for an average of 92.12 fragments of bone per cubic meter of excavated soil. The largest category of bone at this site is Large Fauna, which accounts for 48.58% of the total bone count and over 87% of the weight. This is followed by undifferentiated bone (32.57% by count, 4.28% by weight), small fauna (9.21% by count, 0.67% by weight), and shell (5.26% by count, 5.88% by weight) (**Table S5**). Of the large fauna, 75% had been burned to some degree, and 41.22% was calcined to some degree. Of the undifferentiated bone, 51.52% was burned to some degree, and of that 23.23% was calcined to some degree. Of the small fauna, 25% exhibited some degree of burning, and 21.43% was completely blackened, but none was calcined. One fragment of large fauna bone contains a cut mark from butchering, and one piece appears to have been worked for use as a tool. Three individual bone fragments and one set of articulated bones from this site were taken to the UC Santa Cruz faunal lab for further identification. These include a nearly complete pocket gopher skull, one rib fragment that is from an animal the size of a deer (though lacking clear diagnostic elements), one nearly complete deer rib (bearing cut marks), and tooth fragments from a large herbivore.

Faunal analysis results: Faunal collections indicated similar types of meat consumption and/or preparation at both sites. The majority of the bone came from large fauna, primarily deer based on the identifiable bone, indicating that energy was expended on time-intensive and riskier large game hunting and not organized attempts to take large numbers of small-size but low-risk smaller animals. This indicates that such large game was abundant enough to justify expending time and energy on the high-risk but high-payoff large game, rather than intensifying use of the smaller package-size but more readily available small fauna such as rabbits. This also suggests that the production of meat was a primarily male activity at these sites, possibly organized by individual hunters or small parties and only incidentally engaged in by women and children. The relatively large amount of bone, including small fauna bone that has been burned suggests that while deer and other large game were the focus of consumption, small fauna was also prepared and consumed. This may indicate opportunistic takes of rabbits by hunters, or possibly nearby takes by children and women.

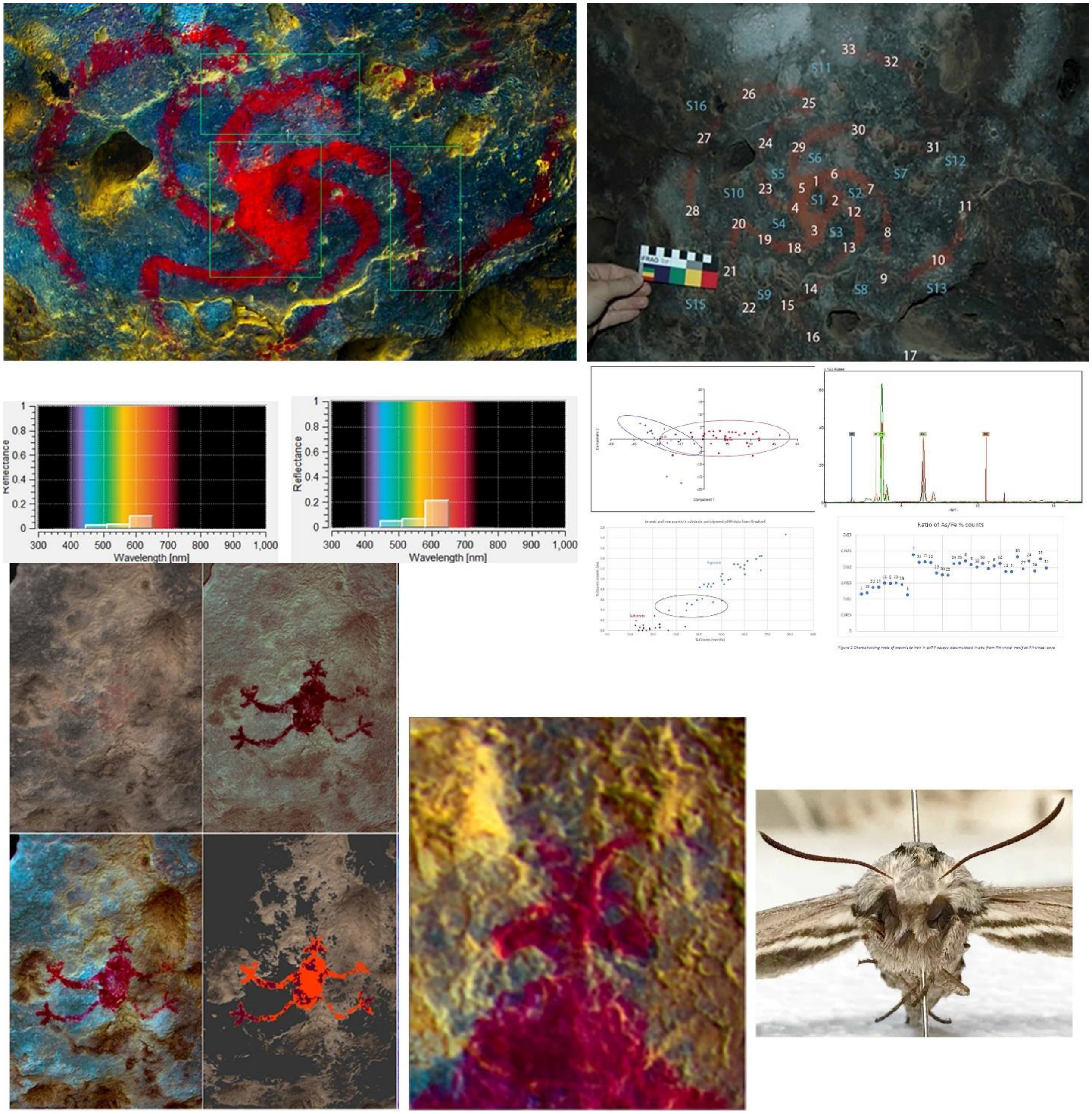

Fig. S1. RTI and pXRF analyses of paintings. Top left, Decorrelation Stretch RTI image with areas of different pigment additions in Pinwheel Painting. **Top right,** locations of pXRF readings in Pinwheel Painting. **Middle left,** spectral reflectance values **taken in** CHER-Ob software from two different areas of the panel, light red (left), dark red (right). **Middle right,** PCA plot of pXRF data from pinwheel motif in Pinwheel cave – sub = substrate, pnw = pigment in pinwheel motif (upper left); XRF spectra from substrate (green) and pigment (red) in point 364 in Pinwheel motif (upper right); Arsenic and iron counts as percentages in substrate and pigment pXRF data. Oval indicates group of points which deviate from the gradient of other pigment data (lower left); Chart showing ratio of arsenic to iron in pXRF assays accumulated in situ from Pinwheel motif (lower right); **Lower left,** RTI image as if lighted from above, decorrelation Stretch images and pseudo-colour image of Transmorph painting; **Lower Middle**, Decorrelation Stretch RTI view of Transmorph head, eye orbits, and antennae; **Lower right;** Hawkmoth specimen, whitelined sphinx moth, *Hyles lineata* (Fabricus). Specimen and photo by David Haviland.

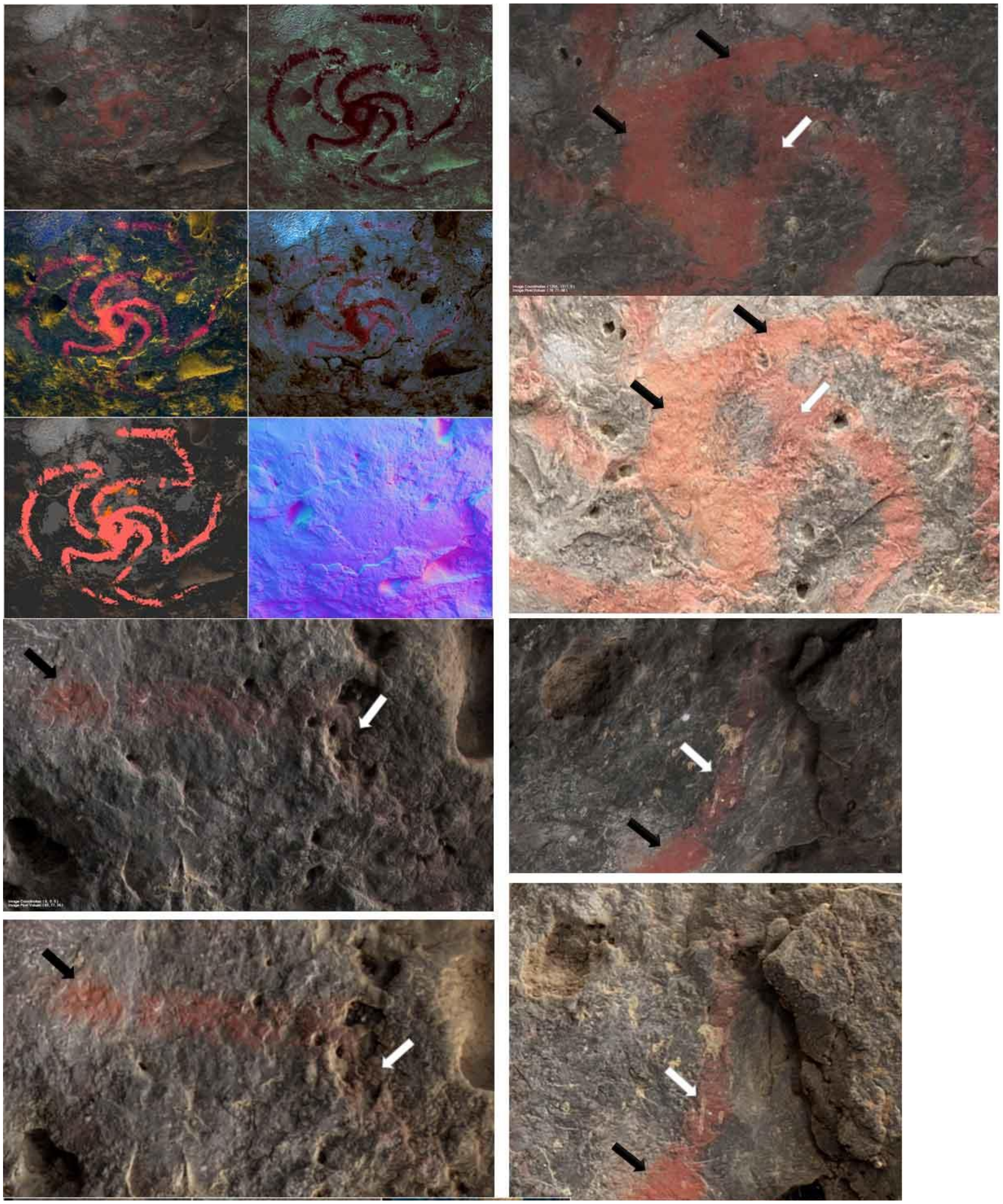

Fig. S2. RTI imaging of Pinwheel element details. Top left, RTI image as if lighted from above and DS image in yre mode (top). DS image in lds and lye mode (middle). Pseudo-colour image and normal map. Top right, RTI rendering of detail 2. Data captured in 2017 (above) and 2015 (below). Black arrows point a lighter red area with a more powdery texture. White arrows point a darker red area, probably painted below. Bottom left, RTI rendering of detail 3. Data captured in 2017 (above) and 2015 (below). Black arrow points a light red area in a better preservation state compared to the darker red area (white arrow). Bottom right, Figure 9: RTI rendering of detail 5. Data captured in 2017 (above) and 2015 (below). Black arrows points a light red area. White arrows point a darker red area.

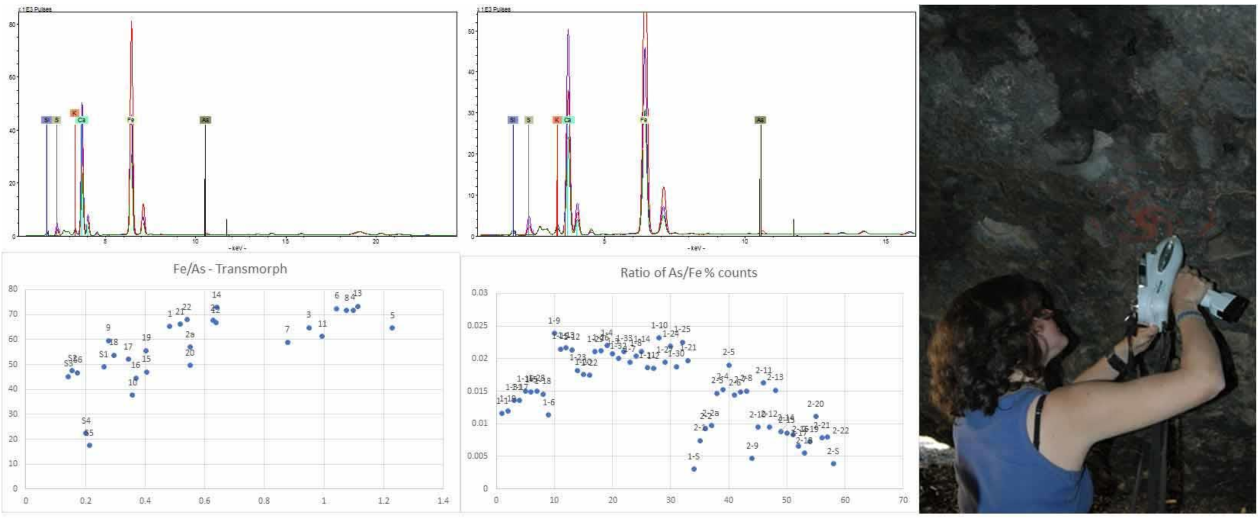

Fig. S3. Analysis of Transmorph with pXRF. ***Top left, PXRF spectra from substrate (green), Tramsmorph body (red) and limb (purple). Top middle, PXRF spectra from substrate (green), Transmorph body (red) and limb (purple). Top right, samples being taken from Pinwheel element. Bottom left, Percentage of iron and arsenic counts in pXRF data from Transmoprh. Bottom right, Ratio of arsenic to iron in pXRF data from Pinwheel motif (1) and the Transmorph (2). Substrate average data are labelled 'S'.***

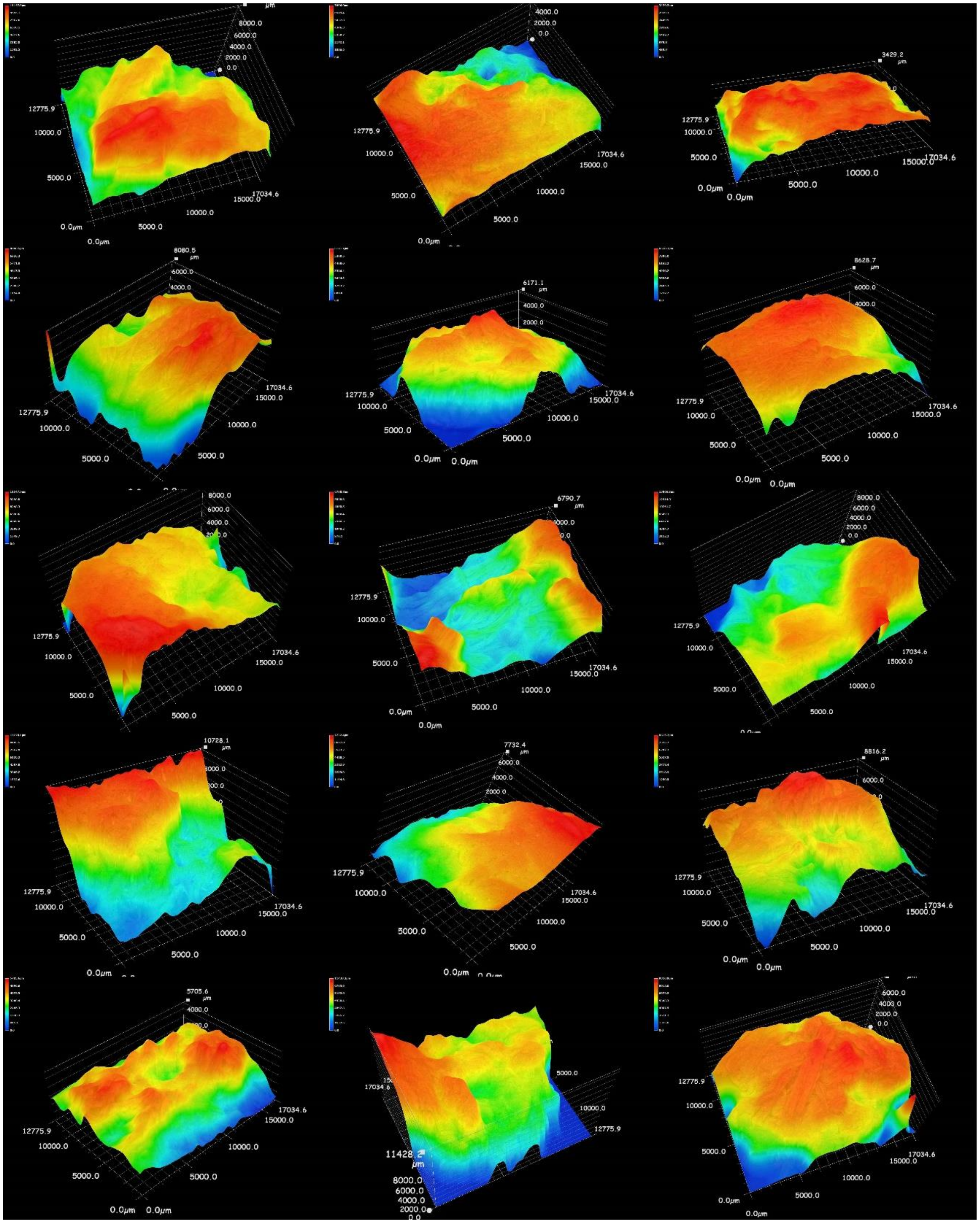

Fig. S4. 3D views of quids. ***A****, Quid 1,* ***B****, Quid 2, Sub-Sample 1,* ***C****, Quid 2, Sub-Sample 3;* ***D****, Quid 2, Sub-Sample 4* ***E****, Quid 2, Sub-Sample 5,* ***F****, Quid 2, Sub-Sample 9* ***G****, Quid B* ***H****, Quid C* ***I*** *Quid E* ***J*** *Quid F1* ***K*** *Quid F2* ***L*** *Quid G1,* ***M*** *Quid G2,* ***N****, Quid H1* ***O****, Quid H2.*

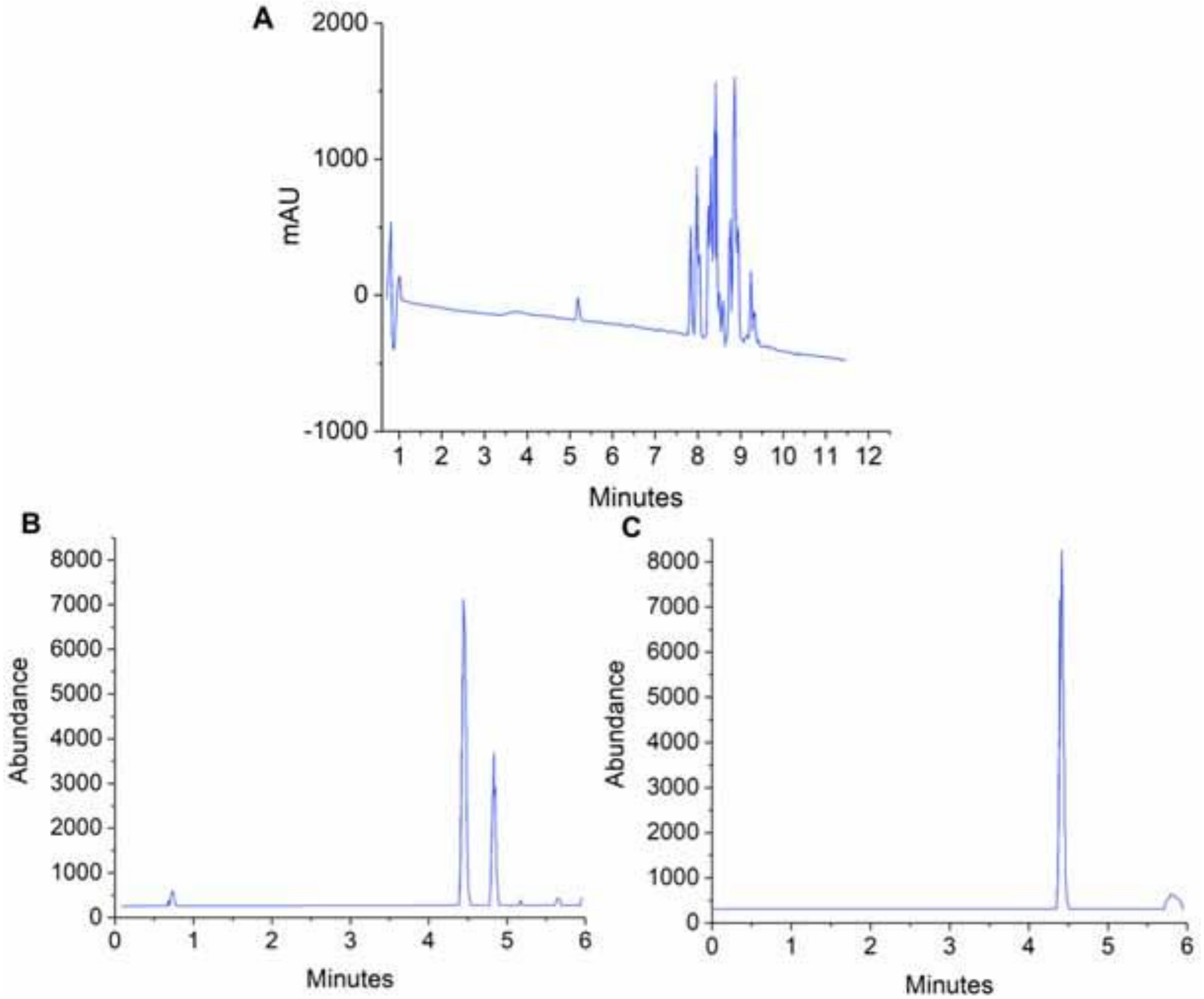

Fig. S5. Analysis of Datura wrightii leaf extract. (**A**) Chromatographic analysis of *Datura* leaf extract at 214 nm. (**B**) EIC of atropine over 290:292 m/z range. (**C**) EIC of scopolamine over 304:306 m/z range.

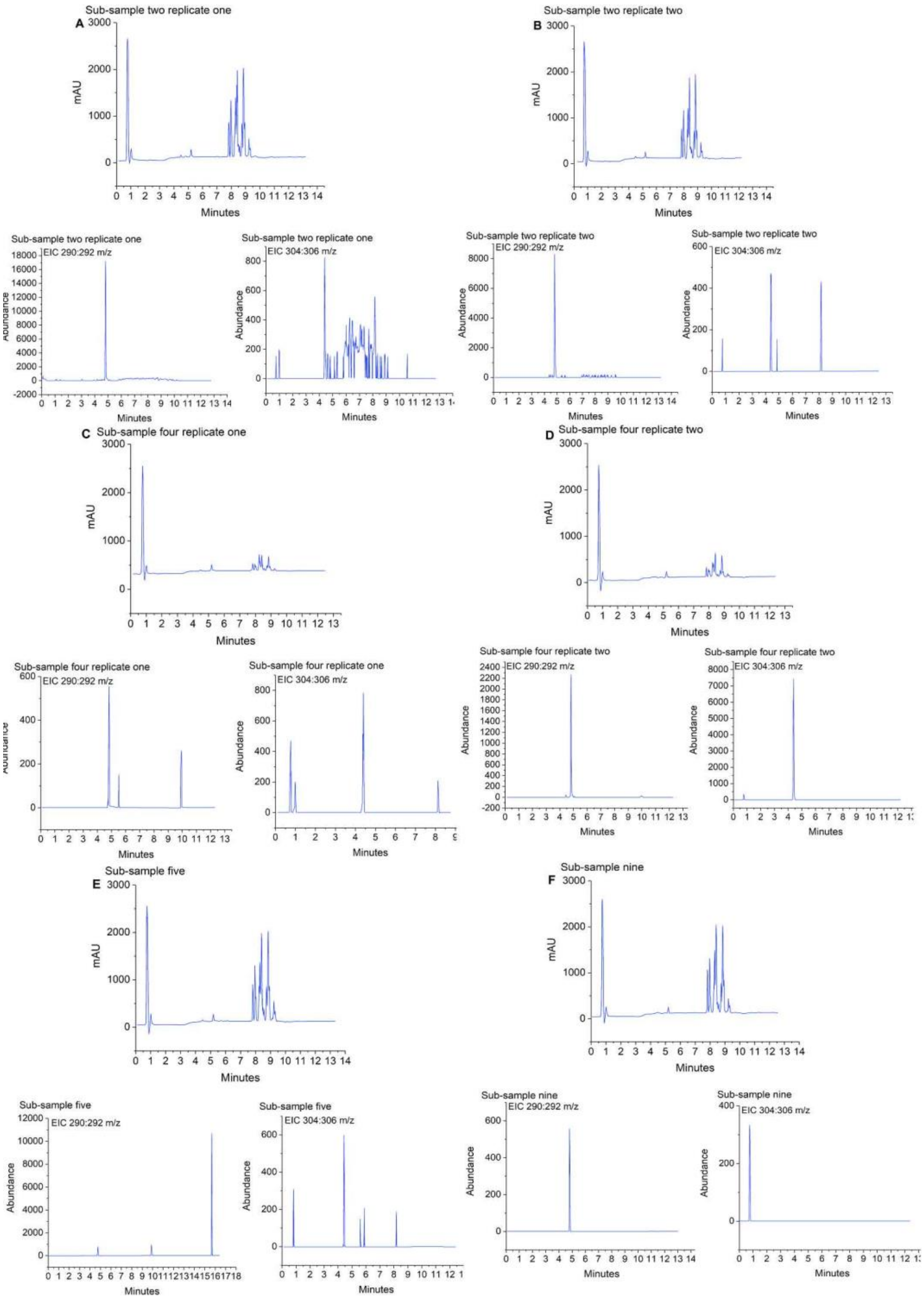

Fig. S6. Chromatographic/EIC analysis from quid extracts. (**A**) Chromatographic analysis and EIC of sub-sample two replicate one. (**B**) Chromatographic analysis and EIC of sub-sample two replicate two. (**C**) Chromatographic analysis and EIC of sub-sample four replicate one. (**D**) Chromatographic analysis and EIC of sub-sample four replicate two. (**E**) Chromatographic analysis and EIC of sub-sample five. (**F**) Chromatographic analysis and EIC of sub-sample nine.

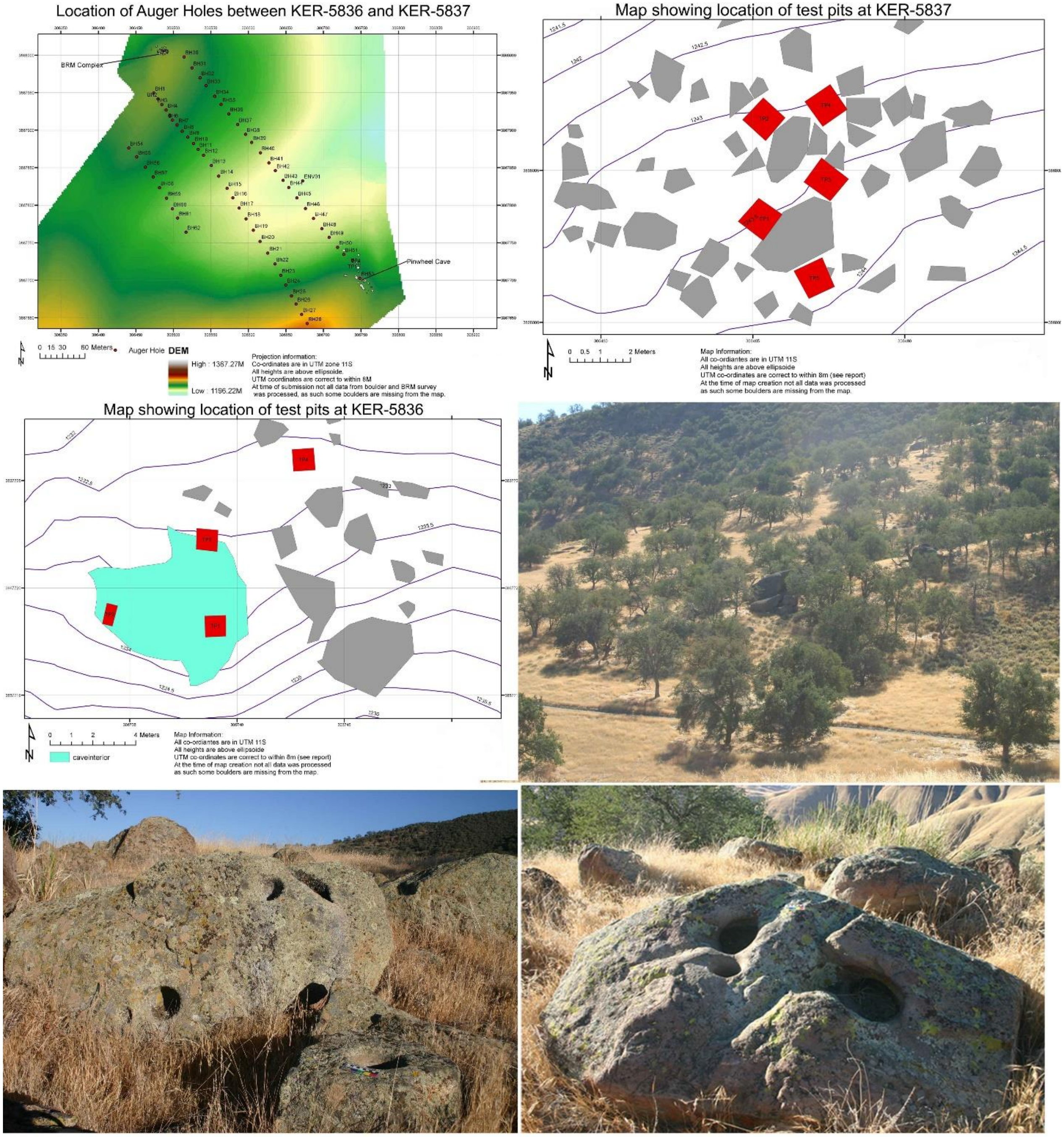

Fig. S7. Archaeological excavations and views of Pinwheel Cave (KER-5836) and Bedrock Mortar complex (KER-5837). Upper left, location of augur samples; **upper right,** map of excavated units at bedrock mortar complex; **middle left,** map of excavated units at Pinwheel Cave; **middle right**, view of Pinwheel Cave formation in oak woodland; **lower left,** view of bedrock mortars; **lower right,** view of bedrock mortars.

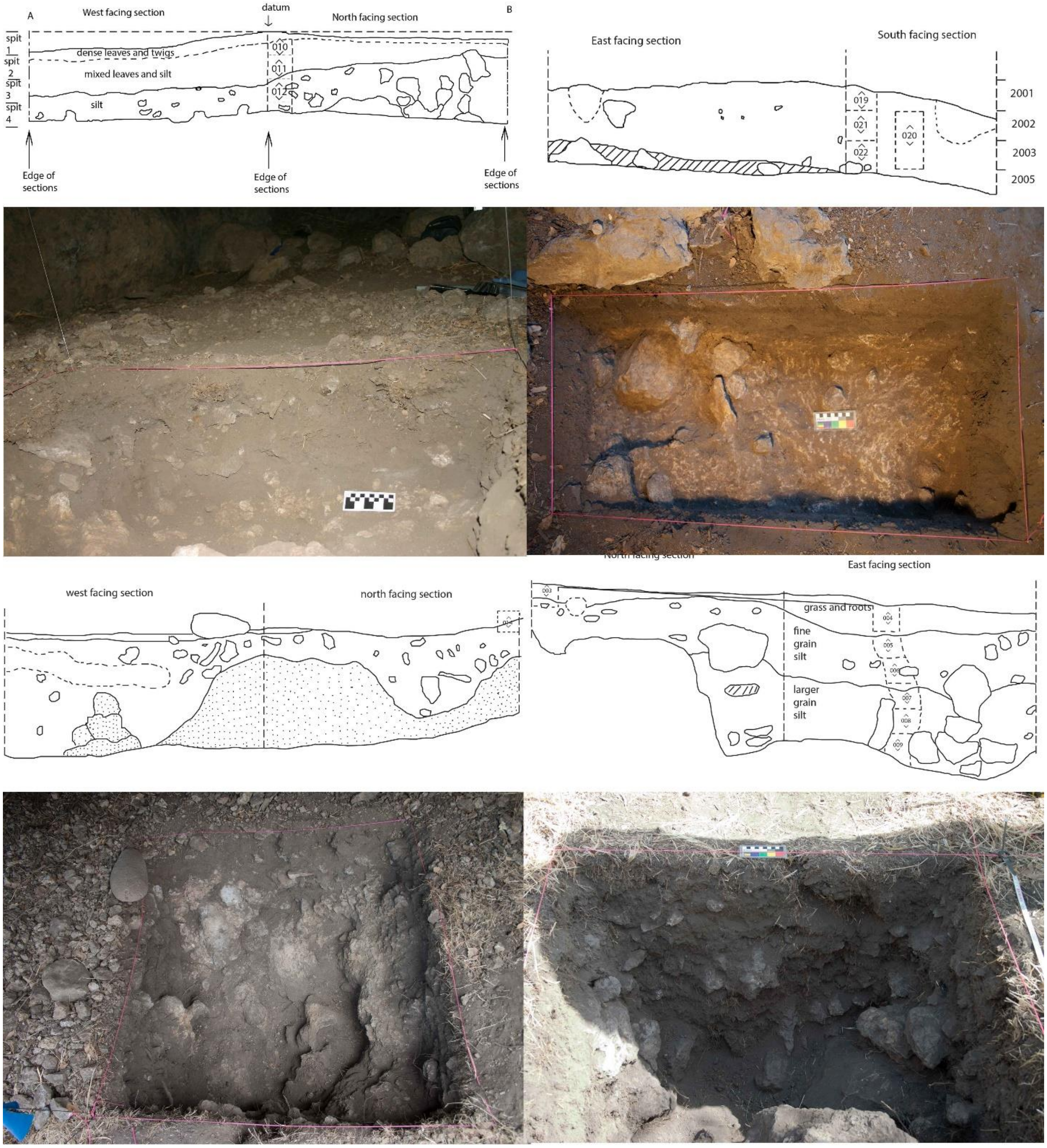

Fig. S8. Archaeological excavations and section drawings of Test Pits at Pinwheel Cave (KER-5836). Upper left, Test Pit 1; upper right, Test Pit 2; lower left, Test Pit 3; lower right, Test Pit 4.

Table S1. Catalog of beads from Pinwheel Cave (KER-5836).

Catalog #	Unit	Level	Materia l	Type	Details	Perforation	Maximum diameter (mm)	Maximum thickness (mm)
116	1	0-10	Shell	*Olivella biplicata* wall		Microdrill	6.8	
120	1	10-20	Shell	*Olivella biplicata* wall		Microdrill	7.01	
144	1	20-30	Shell	*Olivella biplicata* wall	Chipped edge	Microdrill	7.14	
143	1	20-30	Shell	*Haliotis rufescens* disc		Microdrill	4.26	1.35
130	1	20-30	Shell	*Olivella biplicata* wall		Microdrill	7.42	
141	1	20-30	Shell	*Haliotis rufescens* disc		Microdrill	3.73	1.26
129	1	20-30	Glass	Drawn	Burned	Microdrill	6.44	4.38
145	1	30-40	Shell	*Olivella biplicata cuppe*	Top surface grou	Microdrill	3.72	
146	1	30-40	Shell	*Olivella biplicata cupped*		Microdrill	3.28	
147	1	30-40	Shell	*Mytilus californianus tube*		Microdrill	3.34	3.19
140	1	30-40	Shell	*Olivella biplicata* wall		Microdrill	6.34	
5	3	10-20	Glass	Spheroidal freewound		Microdrill		
6	3	10-20	Shell	*Olivella biplicata* wall		Microdrill	3.71	
157	3	10-20	Shell	*Olivella biplicata* wall		Needle-drilled	5.63	
158	3	10-20	Glass	Drawn: D/MCHU*	Blue: Pantone 305		3.67	3.1
40	3	30-40	Shell	*Mytilus californianus* disc		Microdrill	4.47	2.34
41	3	30-40	Shell	*Olivella biplicata* wall	Chipped edge	Needle-drilled	5.21	
42	3	30-40	Shell	*Olivella biplicata* wall	Chipped edge	Microdrill	6.37	
43	3	30-40	Glass	Drawn: D/MCHU*	Blue: Pantone 298		4.39	3.68
14	3	50-60	Shell	*Mytilus californianus* disc		Microdrill	6.96	2.49
110	4	30-40	Shell	*Olivella biplicata* lipped	Full lipped bead	Large; microdril	6.48	
108	4	30-40	Shell	*Mytilus californianus disc*		Microdrill	6.86	3.22
72	4	40-50	Shell	*Olivella biplicata* wall		Microdrill	6.23	
47	4	50-60	Shell	*Olivella biplicata* lipped	Full lipped; oval	Microdrill	10.96	
92	4	60-70	Shell	*Olivella biplicata* lipped	Thin lipped	Large; microdril	5.29	

Table S2. Catalog of beads from Bedrock Mortar Complex (KER-5837).

Catalog #	Unit	Level	Material	Type	Details	Perforation	Maximum diameter (mm)	Maximum thickness (mm)
10	1	20-30	Stone: schisto	Disc	Golden brown-black	Large; microdr	5.75	2.98
37	2	10-20	Shell	*Olivella biplicata* wall	Heavily weathered	Microdrill	3.72	
39	2	10-20	Shell	*Olivella biplicata* wall	Chipped edge; heavily weathere	Needle-drilled?	5.46	
64	3	20-30	Shell	*Olivella biplicata* wall	Heavily weathered	Microdrill	6.07	
73	3	40-50	Shell	*Olivella biplicata* spire-gro	Heavily weathered; trace asphal			6.26: length
15	4	0-10	Shell	*Olivella biplicata* lipped	Thin lipped	Large; microdr	5.65	
97	4	10-20	Glass	Drawn: D/MCHU*	Green: Pantone 339		3.22	3.44
90	4	10-20	Glass	Drawn: D/MCHU*	Blue: Pantone 293		3.28	2.63
88	4	10-20	Shell	*Olivella biplicata* wall	Heavily weathered	Microdrill	6.09	
92	4	10-20	Shell	*Olivella biplicata* wall		Microdrill	6.33	
91	4	10-20	Shell	*Olivella biplicata cupped*		Microdrill	3.65	
89	4	10-20	Shell	Tiv*ela stultorum*; detritus	Heavily weathered			
99	4	20-30	Shell	*Olivella biplicata* wall	Chipped edge	Needle-drilled	5.99	
104	4	20-30	Shell	*Olivella biplicata* wall	Heavily weathered	Microdrill	4.08	
121	4	60-70	Shell	*Olivella biplicata* spire-ground				12.23: length

Table S3. Density of Carbonized Plant Remains at Pinwheel Cave (CA-KER-5836), measured by count (n) and/or weight (mg) per liter (L) soil volume. Both count and weight densities shown for large taxa that break into smaller pieces, whereas only count density presented for small-seeded taxa that typically weigh <1.0 mg.

			**Interior**		**Entrance**		**Exterior**		
**Unit**<br>**Depth (cm) below surface**			**TP1 10-20**	**TP1 30-40**	**TP3 30-40**	**TP3 50-60**	**TP4 10-20**	**TP4 40-50**	**TP4 60-70**
**Taxon**	**Common Name**	**#/L**							
Juniperus sp.	Juniper	n	--	--	1.0	--	--	2.0	--
		mg	--	--	3.0	--	--	5.0	--
Marah sp.	Wild cucumber	n	--	9.0	1.0	1.0	2.0	--	--
		mg	--	<1.0	1.0	5.0	2.0	--	--
Quercus spp.	Acorn nutshell	n	2.0	151.0	48.0	51.0	3.0	--	2.0
		mg	2.0	6.0	4.0	1.0	1.0	--	<1.0
Unid nutshell		n	--	2.0	2.0	1.0	2.0	--	--
		mg	--	1.0	1.0	<1.0	5.0	--	--
Wood Charcoal		mg	150.0	640.0	140.0	420.0	340.0	570.0	40.0
<u>Small Seeded Taxa (n/L)</u>									
Artemesia tridentata	Big Sagebrush	n	--	--	3.0	--	--	--	--
Bromus sp.	Brome grass	n	--	4.0	--	--	--	--	--
Chenopodium berlandieri	Goosefoot	n	--	4.0	4.0	3.0	--	--	--
Claytonia spp.	Miners Lettuce	n	--	--	1.0	1.0	--	--	--
Deschampsia spp.	Hairgrass	n	--	--	1.0	--	--	--	--
Erodium sp.	Filaree	n	--	--	--	1.0	--	--	1.0
Hordeum spp.	Wild Barley	n	--	2.0	--	1.0	--	--	--
Juncus spp.	Indian Rush	n	--	4.0	--	4.0	--	--	--
Mentzelia spp.	Blazing Star	n	--	8.0	--	--	--	--	--
Papaver sp.	Fire Poppy	n	--	12.0	6.0	4.0	3.0	--	--
Phacelia spp.	Phacelia	n	--	--	3.0	1.0	--	--	--
Poa sp.	Bluegrass	n	--	--	14.0	--	--	--	27.0
Potamogeton spp.	Pondweed	n	--	--	1.0	--	--	--	--
Salvia columbariae	Chia	n	--	--	3.0	2.0	1.0	1.0	1.0
Sambucus sp.	Elderberry	n	--	4.0	2.0	1.0	--	--	--
Schoenoplectus acutus	Tule	n	--	1.0	2.0	--	--	--	--
Silene spp.	Catchfly	n	--	--	--	3.0	1.0	--	--

Sporobolus	Alkali Sacaton	n	--	--	--	--	--	--	1.0
Trifolium spp.	Clover	n	--	1.0	2.0	6.0	1.0	1.0	1.0
Typha spp.	Cattail	n	12.0	28.0	5.0	1.0	--	--	--
Veronica sp.	Speedwell	n	--	--	--	--	--	--	16.0
Vulpia	Fescue Grass	n	--	--	3.0	2.0	--	2.0	1.0
Asteraceae	Sunflower Family	n	--	--	4.0	3.0	--	1.0	--
Borageaceae	Borage Family	n	--	--	--	--	--	1.0	--
Brassicaceae	Mustard Family	n	--	--	--	1.0	--	--	--
Cactaceae	Cactus Family	n	--	--	1.0	--	--	--	--
Fabaceae	Bean Family	n	--	--	8.0	4.0	--	--	--
Malvaceae	Mallow Family	n	--	--	--	1.0	--	--	--
Poaceae	Grass Family	n	4.0	87.0	91.0	166.0	36.0	44.0	26.0
Monocot Stem Frags		n	17.0	32.0	4.0	3.0	--	2.0	--
UnID embryos		n	--	1.0	3.0	3.0	--	--	--
UnID seeds		n	--	25.0	18.0	11.0	12.0	--	31.0
UnID seed fragments		n	16.0	45.0	22.0	22.0	23.0	4.0	3.0

Table S4. Density of Carbonized Plant Remains at the Bedrock Mortar (BRM) Site (CA-KER-5837), measured by count (n) and/or weight (mg) per liter (L) soil volume. Both count and weight densities shown for large taxa that break into smaller pieces, whereas only count density presented for small-seeded taxa that typically weigh <1.0 mg.

**Unit**<br>**Depth (cm) Below Surface**<br><br>**Taxon**	**Common Name**	**#/L**	**TP1**<br>**20-30**	**TP1**<br>**30-40**	**TP4**<br>**20-30**	**TP4**<br>**40-50**
Juniperus sp.	Juniper	n	5.0	10.0	6.0	6.0
		mg	3.0	2.0	1.0	6.0
Marah sp.	Wild cucumber	n	--	--	5.0	--
		mg	--	--	2.0	--
Quercus spp.	Acorn nutshell	n	4.0	4.0	1.0	--
		mg	1.0	<1.0	1.0	--
Unid nutshell		n	--	--	1.0	1.0
		mg	--	--	5.0	1.0
Wood Charcoal		mg	540.0	340.0	190.0	960.0
<u>Small Seeded Taxa (n/L)</u>						
Chenopodium berlandieri	Goosefoot	n	1.0	1.0	2.0	--
Deschampsia spp.	Hairgrass	n	2.0	1.0	--	--
Erodium sp.	Filaree	n	--	--	--	1.0
Galium spp.	Bedstraw	n	--	--	--	1.0
Hordeum spp.	Wild Barley	n	4.0	1.0	--	--
Juncus spp.	Indian Rush	n	--	--	1.0	1.0
Mentzelia spp.	Blazing Star	n	--	--	2.0	1.0
Papaver sp.	Fire Poppy	n	--	1.0	--	--
Phacelia spp.	Phacelia	n	4.0	1.0	--	1.0
Plantago spp.	Plantain	n	--	--	--	1.0
Poa sp.	Bluegrass	n	--	--	1.0	--
Salvia columbariae	Chia	n	1.0	2.0	3.0	2.0
Sambucus sp.	Elderberry	n	4.0	3.0	1.0	3.0
Typha spp.	Cattail	n	--	3.0	--	--
Asteraceae	Sunflower Family	n	4.0	5.0	1.0	6.0
Fabaceae	Bean Family	n	--	3.0	2.0	2.0
Papaveraceae	Poppy Family	n	--	--	1.0	--
Poaceae	Grass Family	n	58.0	37.0	52.0	82.0
Solanaceae	Nightshade Family	n	--	1.0	--	--
Monocot Stem Frags		n	--	3.0	--	--
UnID embryos		n	--	--	2.0	--
UnID seeds		n	13.0	7.0	--	--
UnID seed fragments		n	43.0	16.0	18.0	18.0

Table S5. Faunal remains from Pinwheel Cave (KER-5836) and the Bedrock Mortar site (KER-5837).

	Pinwheel Cave fauna				BRM complex fauna			
	Count	% Count	Weight	% Weight	Count	% Count	Weight	%Weight
**Bird**	**2**	**0.19%**	**0.1**	**0.02%**	**2**	**0.66%**	**0.3**	**0.40%**
**Burrowing Rodent**	**45**	**4.30%**	**2.2**	**0.54%**	**7**	**2.30%**	**0.4**	**0.53%**
**Fish**	**26**	**2.49%**	**0.9**	**0.22%**	**2**	**0.66%**	**0.0**	**0.00%**
**Large Fauna**	**339**	**32.41%**	**366.4**	**90.56%**	**148**	**48.68%**	**65.7**	**87.83%**
**Medium Fauna**	**6**	**0.57%**	**2.6**	**0.64%**		**0.00%**		**0.00%**
**Med-Lg. Fauna**	**4**	**0.38%**	**2.3**	**0.57%**	**1**	**0.33%**	**0**	**0.00%**
**Reptile/Amphibian**	**16**	**1.53%**	**1.2**	**0.30%**	**1**	**0.33%**	**0.3**	**0.40%**
**Shell**	**55**	**5.26%**	**1.9**	**0.47%**	**16**	**5.26%**	**4.4**	**5.88%**
**Sm-Med Fauna**	**10**	**0.96%**	**1.1**	**0.27%**		**0.00%**		**0.00%**
**Small Fauna**	**246**	**23.52%**	**14.5**	**3.58%**	**28**	**9.21%**	**0.5**	**0.67%**
**Undif. Bone**	**297**	**28.39%**	**11.4**	**2.82%**	**99**	**32.57%**	**3.2**	**4.28%**
Total	1046		404.6		304		74.8	

Table S6. AMS dates from Pinwheel Cave (KER-5836) and the Bedrock Mortar site (KER-5837).

Lab #	Location	Depth	14C Age	14C Error	Material	ΔR	ΔR Error	2σ cal BP Unmodeled	2σ cal BP Modeled	2σ cal BC/AD Modeled
PSUAMS-6264	Cave Ceiling	N/A	140	15	Quid B	N/A	N/A	275-10	280-85	AD 1670-1865
PSUAMS-6265	Cave Ceiling	N/A	105	20	Quid C	N/A	N/A	265-25	270-85	AD 1680-1865
PSUAMS-6266	Cave Ceiling	N/A	210	15	Quid E	N/A	N/A	300-0	300-150	AD 1650-1800
PSUAMS-6267	Cave Ceiling	N/A	145	15	Quid F	N/A	N/A	280-5	285-110	AD 1665-1840
PSUAMS-6268	Cave Ceiling	N/A	230	20	Quid G	N/A	N/A	305-0	305-155	AD 1645-1795
PSUAMS-6269	Cave Ceiling	N/A	295	15	Quid H	N/A	N/A	430-300	420-295	AD 1530-1655
SUERC-44589	Cave Ceiling	N/A	239	29	Quid 1 - Daturra sp.	N/A	N/A	425-0	315-150	AD 1635-1800
SUERC-44590	Cave Ceiling	N/A	114	29	Quid 2 - Daturra sp.	N/A	N/A	270-10	280-90	AD 1670-1860
SUERC-46061	Pinwheel Cave, TP3	40-50 cm	212	29	Charcoal	N/A	N/A	305-0	305-140	AD 1645-1810
SUERC-29934	Pinwheel Cave, TP4	30-40 cm	810	35	*O. biplicata* Bead	317	42	240-0	265-90	AD 1685-1860
SUERC-46062	Pinwheel Cave, TP1	20-30 cm	80	29	Mammal Bone	N/A	N/A	260-25	240-60	AD 1710-1890
SUERC-29935	Pinwheel Cave, TP1	20-30 cm	795	35	*O. biplicata* Bead	309	42	240-0	235-60	AD 1715-1890
SUERC-29937	Bedrock Mortar, TP4	60-70 cm	845	35	*O. biplicata* Bead	324	42	260-0	380-105	AD 1570-1845
SUERC-29936	Bedrock Mortar, TP4	20-30 cm	1000	35	*O. biplicata* Bead	347	42	460-150	345-85	AD 1605-1865

Movie S1 (separate file). Fly through of Pinwheel Cave laser-scan point cloud.

SI References

1. M. Mudge, T. Malzbender, C. Schroer, and M. Lum. New Reflection Transformation Imaging Methods for Rock Art and Multiple-Viewpoint Display. *VAST* **6**, 195-202 (2006).
2. E. Kotoula, G. Earl, (2014). Integrated RTI approaches for the study of painted surfaces. *Comp. Applications Quant. Meth. Archaeol.* 22-25 (2014).
3. E. Kotoula, D. W. Robinson, C. Bedford, Interactive relighting, digital image enhancement and inclusive diagrammatic representations for the analysis of rock art superimposition: The main Pleito cave (CA, USA). *J. Archaeol. Sci.* ***93***, 26-41 (2018).
4. J. Harman, DStretch. http://www.dstretch.com/AlgorithmDescription.html, (2008).
5. M. Gil, M.L. Carvalhob, A. Seruya, A.E. Candeiasd, J. Miraoe, I. Queralt. Yellow and red ochre pigments from southern Portugal: Elemental composition and characterization by WDXRF and XRD *Nuclear Instru. Meth. Physics Research A* ***580**, 728-731 (2007)*
